# New insights into the base catalyzed depolymerization of technical lignins: a systematic comparison†

**DOI:** 10.1039/d2ra06998a

**Published:** 2023-02-08

**Authors:** Rajeesh Kumar Pazhavelikkakath Purushothaman, Gijs van Erven, Daan S. van Es, Léon Rohrbach, Augustinus E. Frissen, Jacco van Haveren, Richard J. A. Gosselink

**Affiliations:** a Wageningen Food & Biobased Research Bornse Weilanden 9 6708 WG Wageningen The Netherlands richard.gosselink@wur.nl rajeesh.pazhavelikkakathpurushothaman@wur.nl; b Wageningen University & Research, Laboratory of Food Chemistry Bornse Weilanden 9 6708 WG Wageningen The Netherlands; c Green Chemical Reaction Engineering, ENTEG, University of Groningen Nijenborgh 4 9747 AG Groningen the Netherlands

## Abstract

A first systematic approach on the base catalyzed depolymerization (BCD) of five technical lignins derived from various botanical origins (herbaceous, hardwood and softwood) and covering the main three industrial pulping methods (soda, kraft and organosolv) is reported. This study provides a first of its kind in-depth quantification and structural characterization of two main BCD fractions namely lignin oil and lignin residue, describing the influence of the BCD process conditions. Depolymerization is evaluated in terms of lignin conversion, lignin oil yield, phenolic monomer selectivity and the production of lignin residue and char. Lignin oils were extensively characterized by size exclusion chromatography (SEC), GC-MS, GC-FID, ^13^C-NMR, HSQC NMR and elemental analysis. GC × GC-FID was used to identify and quantify distinct groups of monomeric compounds (methoxy phenols, phenols, dihydroxy-benzenes) in the lignin oil. The lignin oil yields (w/w) ranged from 20–31% with total monomer contents ranging from 48 to 57% w/w. SEC analysis indicated the presence of dimers/oligomers in the lignin oil, which through HSQC NMR analysis were confirmed to contain new, non-native interunit linkages. ^13^C NMR analyses of the lignin oils suggest the presence of diaryl type linkages (*i.e.* aryl–aryl, aryl C–O) evidencing deconstruction and recombination of lignin fragments during BCD. Irrespective of the lignin source, a residue, often regarded as ‘unreacted’ residual lignin was the main product of BCD (43 to 70% w/w). Our study highlights that this residue has different structural properties and should not be considered as unreacted lignin, but rather as an alkali soluble condensed aromatic material. HSQC, DEPT-135, ^13^C, and ^31^P NMR and SEC analyses confirm that the BCD residues are indeed more condensed, with increased phenolic hydroxyl content and lower molecular weights compared to all feed lignins. Subsequent BCD of solid residual fractions produced only low oil yields (6–9% w/w) with lower phenolic monomer yields (4% w/w) compared to original lignin, confirming the significantly more recalcitrant structure. Our study improves the overall understanding of the BCD process, highlights important feedstock-dependent outcomes and ultimately contributes to the complete valorization of BCD-derived lignin streams.

## Abstract 2

Towards a lignin BCD biorefinery! Overall understanding of the BCD process, feedstock-dependent fractionation results, complete valorization of BCD-derived lignin streams.

## Graphical abstract

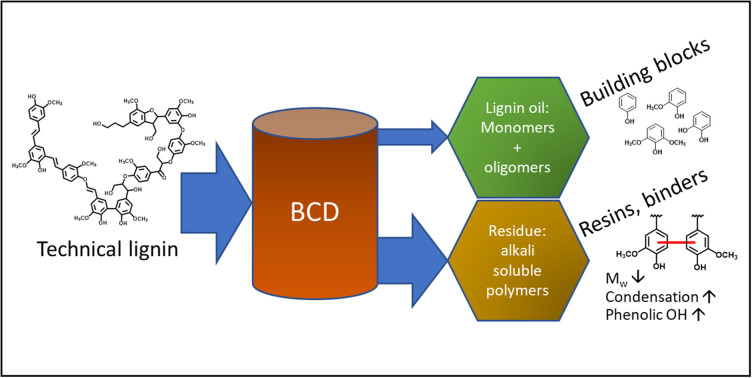

## Introduction

According to the 2015 Paris Climate Agreement, global warming needs to be restricted to 1.5 °C to reduce the impacts of climate change.^1^ In the EU, the aim is to reduce GHG emission of at least 55% compared to the 1990 level by 2030, concurrently with a reduction in fossil based chemicals and fuels consumption.^2^ Lignocellulosic residues and side streams are excellent sources of renewable carbon due to their large availability and potentially net zero greenhouse gas emissions.^3–5^ Progressive developments have been made on the conversion of lignocellulosic biomass to renewable chemicals, energy and fuels, but still mainly the (hemi)cellulosic fractions are targeted. Lignin, the second most abundant fraction of lignocellulosic biomass, represents a renewable source for aromatic monomers with potential to replace fossil based resources.^6^

Lignocellulosic biorefineries such as cellulosic ethanol, sugar hydrolysate and paper mill industries are producing a surplus of lignin residues, that are often simply burned for energy recovery. However, there is an increasing trend to explore the upgrading of lignin residues to higher value products, to contribute to circular carbon economy.^7,8^ Lignin is a heterogenous complex aromatic polymer consisting of methoxylated phenyl propane units connected by various C–C and C–O–C bonds, of which the β-O-4 aryl ether is most abundant.^9^ As such, lignin could provide an excellent source of bio-based aromatic monomers, mainly phenolics. Lignin-derived monomeric phenolic compounds are potential renewable candidates for various industrial applications, *e.g.* building blocks for polymers, resins or as a fuel component.^6^ However, production of industrially relevant aromatic base chemicals from the heterogenic and recalcitrant lignin polymer requires selective deconstruction or depolymerization of the lignin structure and often chemical upgrading in terms of fractionation or further conversion is needed to meet the requirements of the intended application. Despite significant advances in the field of reductive catalytic fractionation (RCF),^10^ producing phenolic monomers directly from lignocellulosic biomass, conventional pulp mills are anticipated to continue to operate and thus will provide a continuous stream of technical lignins to be upgraded.^11,12^

Several depolymerization methods have been investigated for native and industrial lignins.^6,13,14^ Native lignin produced by mild lignocellulosic fractionation can be catalytically depolymerized more easily through β-O-4 cleavage. However, during industrial pulping native lignin bonds are mostly broken and altered resulting in more recalcitrant technical lignins.^15^ Thus, methods developed for native lignin depolymerization are often not suitable for technical lignins. The most common depolymerization methods for technical lignin to aromatics include microbial enzymatic methods,^16–18^ catalytic pyrolysis,^19–21^ base catalyzed depolymerization (BCD),^22–27^ catalytic hydrotreatment,^28,29^ oxidative cleavage.^30^ Commercialization of enzymatic lignin depolymerization is limited due to high enzyme cost and low productivity.^18^ Pyrolysis is an attractive technology, without extensive use of solvents. However, catalytic pyrolysis of lignin and subsequent upgrading of bio-oil to aromatics is non-selective and requires high temperatures and high hydrogen pressures.^21,31,32^ Catalytic hydrotreatment of lignin to produce aromatics has been studied extensively. Such catalytic hydrotreatment has predominantly been carried out in alcoholic solvents (methanol, ethanol) under supercritical conditions or in hydrothermal melt conditions.^20,33,34^ During such catalytic processes, depolymerization of lignin, hydrodeoxygenation and alkylation of the phenolics may occur, leading to a mixture of a number of alkylated aromatics with varying oxygen content.^35–38^ Adversely, the alcohol solvents are not inert during the hydrotreatment reaction and are converted into gaseous products, like ethers, and are partially incorporated into the lignin structure leading to a solvent inefficient process, which is currently being overlooked by most researchers. To obtain high purity monomers such as phenol, further dealkylation and hydrodeoxygenation steps are required. Upgrading *via* catalytic hydrodeoxygenation or hydrogenolysis is often accompanied by ring hydrogenation reactions leading to the formation of cycloaliphatic derivatives.

Base catalyzed deconstruction or depolymerization (BCD) is a well-known method for the depolymerization of lignin under aqueous conditions in the presence of a homogeneous base (*e.g.*, NaOH, KOH, LiOH).^22,39–41^ The base acts as a catalyst, and ensures complete solubilization of the lignin in the aqueous phase. BCD is generally applied in hot compressed alkaline water at a temperature range of 200–300 °C for a reaction time between 30–240 minutes,^42^ resulting in the scission of C–O linkages between lignin units to form phenolic monomers and oligomers. Several papers on BCD of lignin have already appeared in the last decades, showing that substantial amounts of lignin oil, with decent amounts of monomeric phenolics can be produced.^22,23,25,40,41,43,44^ However, BCD processes reported in the literature have primarily focused on optimizing the bulk lignin oil yields, rather than assessing the conversion into specific phenolic components (such as phenols, guaiacol, catechol, syringol *etc.*) or species varying in aromatic ring substitution. Evidently, the process can be further evaluated and optimized in terms of effectivity and selectivity. Furthermore, the literature currently lacks a systematic evaluation of batch BCD of various industrial lignins with respect to botanical origin and pulping processes. Importantly, despite being the most abundant fraction recovered after BCD, surprisingly little attention has thus far been paid to the characterization of these base solubilized lignin residue fractions. Further, a clear description of conversion and yield of phenolics (such as phenol, guaiacol, catechol, syringol *etc.*) relative to the lignin intake was not shown. Besides technical lignin from the pulp and paper industry, BCD was also applied to lignin streams obtained by other biorefinery processes, reporting lignin residues as the main product.^23^ BCD in super critical conditions under continuous flow set-up resulted in low phenolic monomers and high heavy oligomeric lignin oil yields due to the incomplete depolymerization.^26,45,46^ Nonetheless, lignin residue was obtained as the main fraction in both batch and continuous flow BCD reaction conditions, that was largely overlooked by the researchers. Lignin residue is often considered as the non-depolymerized lignin fragments with anticipated structural similarity to the feed lignin. To the best of our knowledge, a clear definition and structural characterization of lignin residue is not available in the literature. Additionally, detailed characterization of BCD lignin oil, especially the nature of the oligomeric fraction is still lacking in the literature. Mattsson *et al.* showed by 2D NMR that lignin oil obtained from LignoBoost™ kraft lignin in supercritical water consisted of phenolic monomers and re-polymerized oligomers with new structural networks.^47^ For a better understanding, complete lignin valorization and cost effectiveness of BCD process of various technical lignins from different botanical origins in aqueous base conditions, an additional structural characterization of BCD fractions is still necessary.

Here, we report a systematic comparison of BCD performed on five industrially relevant technical lignins. We compare mass balances (lignin oil, residue, char), lignin oil yields, monomer yields and selectivity both in terms of specific phenolics as well as compound classes, and in addition comprehensively characterize the lignin residues and lignin oil. To that end, we applied an extensive suit of analytical tools, including GC-MS, GCxGC-FID, SEC, ^13^C NMR, HSQC NMR, ^31^P NMR and elemental analyses. Our results contribute to improving the overall understanding of the BCD process, highlight important feedstock-dependent outcomes and pave the way for complete valorization of BCD-derived lignin streams.

## Results and discussion

### Characterization of input (feed) technical lignin samples

Base catalyzed depolymerization (BCD) was performed on five different types of industrially relevant, technical lignins from various botanical origins (wheat straw/sarkanda grass, hardwood and softwood) and covering the main three industrial pulping methods (soda, kraft and organosolv). Prior to BCD, all lignins used in this study were extensively characterized for their chemical composition, molecular weight distribution, detailed structural features, hydroxyl contents and elemental (C, H, O) composition (Tables 1 and 2).

**Table 1:** Lignin composition analysis on dry weight basis^a^

Lignin	Average polysaccharide contents (wt%)					AIL^b^ (wt%)	ASL (wt%)	Ash (wt%)	Total (wt%)	Element (wt%) based on dry weight		
	Arabinan	Xylan	Mannan	Galactan	Glucan					C	H	O
Soda P1000	0.4	1.6	0.02	0.2	0.6	84.6	5.5	2.5	95.4	64.2	5.7	28.3
Kraft Indulin AT	0.3	0.6	0.04	0.7	0.1	90.3	1.8	2.6	96.4	63.4	5.7	28.2
OS-Alcell	0.0	0.2	0.03	0.02	0.1	94.3	1.9	<0.1	96.6	67.2	5.9	27.2
OS-spruce	0.01	0.2	0.04	0.03	0.2	95.2	0.7	<0.1	96.4	68.2	5.8	27.4
OS-wheat straw	0.2	0.4	0.6	0.1	0.4	93.0	2.4	<0.1	97.1	66.3	6.0	28.2

a OS = organosolv; AIL = acid-insoluble lignin; ASL = acid-soluble lignin.

b Corrected for ash.

**Table 2:** Detailed structural characterization of technical lignins

	Lignin	P1000	Indulin AT	Alcell	Organosolv-WS	Organosolv-S
	Botanical origin	Wheat straw sarkanda grass	Softwood	Hardwood	Wheat straw	Spruce
	Pulping process	Soda	Kraft	Organosolv	Organosolv	Organosolv
^1^H–^13^C HSQC NMR	**Subunit composition (molar%)**					
	H	6.6	2.8	0.0	2.4	0.6
	S	36.0	0.0	57.0	33.6	0.0
	G	48.0	91.5	35.5	60.4	92.9
	G Cα-ox	3.8	5.8	1.3	1.6	6.5
	S Cα-ox	5.6	0.0	6.2	2.0	0.0
	Total Cα-ox	9.4	5.8	7.5	3.6	6.5
	S/G excluding Cα-ox	0.75	0.00	1.61	0.56	0.00
	S/G including Cα-ox	0.80	0.00	1.72	0.58	0.00
	**Interunit linkages (per 100 Ar)**					
	β-O-4 Aryl ether	8.8	7.0	7.8	11.1	0.7
	β-5 Phenylcoumaran	0.9	1.8	1.8	3.7	3.8
	β–β Resinol	0.7	1.2	2.8	0.7	0.8
	β-O-4 α-Oxidized	0.0	0.0	0.0	0.8	0.0
	β-O-4 α-Ethoxylated	0.0	0.0	2.3	0.0	1.9
	*E*-Enol ether	0.0	2.1	0.0	0.0	0.0
	*Z*-Enol ether	0.0	0.7	0.0	0.0	0.0
	β-5 Stilbene	1.7	8.1	0.0	0.0	4.0
	β-1 Stilbene	2.5	4.4	1.4	0.0	1.4
	Secoisolariciresinol	0.0	1.8	0.0	0.0	0.0
	Epiresinol	0.7	0.7	1.8	0.0	4.0
	G1–G5/G1–G1	1.7	7.1	1.1	1.9	3.0
	Total	17.0	34.8	19.0	18.2	19.7
	Total native	10.4	10.0	12.5	15.5	5.3
	**End-units (per 100 Ar)**					
	Cinnamyl alcohol	0.7	0.9	0.0	0.0	0.0
	Cinnamaldehyde	0.0	0.0	0.0	0.0	0.6
	Dihydrocinnamyl alcohol	0.4	3.3	0.2	0.0	2.0
	Arylglycerol	0.0	0.8	0.0	0.0	0.0
	Benzaldehyde	0.3	0.0	0.4	0.0	0.1
	Hibbert ketone	0.0	0.0	0.0	2.9	1.1
	**Hydroxycinnamic acids (per 100 Ar)**					
	Ferulate	0	0	0	4.2	0
	*p*-Coumarate	0	0	0	3.8	0
	**Flavonoids (per 100 Ar)**					
	Tricin	0	0	0	10.3	0

SEC	*M*_w_ (g mol^−1^)	2520	3530	2250	2350	2600
	*M*_n_ (g mol^−1^)	695	928	933	897	1006
	*Đ*	3.6	3.5	2.4	2.6	2.6

^31^P NMR	**OH content (mmol g^−^** ^**1**^ **)**					
	Aliphatic	1.6	2.1	1.1	2.2	1.4
	Carboxylic acid	1.0	0.4	0.3	0.5	0.1
	Syringyl	0.6	0.0	1.1	0.6	0.0
	Condensed guaiacyl	0.7	1.3	0.8	0.8	1.2
	Guaiacyl	0.7	1.6	0.7	1.1	1.5
	*p*-Hydroxyphenyl	0.5	0.2	0.2	0.5	0.1
	Total phenolic OH	2.5	3.1	2.8	3.0	2.8

The chemical composition of various technical lignins are in accordance with literature (Table 1).^48–50^ The total lignin content (acid insoluble and acid soluble) was higher for organosolv lignins compared to soda and kraft. In general, organosolv lignins were found to be purer compared to soda and kraft lignins, both in terms of residual carbohydrate and ash contents.

Results of the detailed structural characterization of the technical lignins are summarized in Table 2. Since our earlier report on the technical lignins used in this study,^48^ a wealth of information has become available on their substructures and their respective analysis by HSQC NMR,^51,52^ now allowing more in-depth characterization and structural comparison. As quantified by HSQC NMR (for spectra see ESI, Fig. S7–S11†), the initial lignin feedstocks showed typical subunit compositions for their botanical origins, with the softwood lignins (Indulin AT and organosolv-S) being entirely composed of guaiacyl units, herbaceous lignins (soda-P1000 and organosolv-WS) composed of mixed guaiacyl and syringyl units with traces of *p*-hydroxyphenyl units and the hardwood lignin (Alcell) being enriched in syringyl units. The starting technical lignins differed not only in subunit composition, including substantial amounts of Cα-oxidized moieties, but also consisted of entirely different interunit linkage motifs, as a result of the different nature and severity of the processing applied. Compared to other technical lignins, kraft Indulin AT showed a significant abundance of enol ether and stilbene sub-units. In addition, the organosolv spruce and Alcell lignins contained appreciable amounts of α-ethoxylated β-O-4 units. Although in low amounts, β-O-4 was the most abundant detectable interunit linkage in all the technical lignins except for organosolv spruce. In the latter only a minor amount is detected.

Besides interunit linkages, the lignins differed substantially in the type and number of end-units. In this context, the presence of Hibbert ketones in the wheat straw and spruce organosolv lignins deserves to be mentioned, and these are indicative of acid-catalyzed depolymerization. Both softwood lignins, Indulin AT and organosolv spruce, contained significant amounts of dihydrocinnamyl alcohol moieties. The organosolv wheat straw lignin particularly differed from the others by having both the hydroxycinnamic acids ferulate and *p*-coumarate as well as the flavonoid tricin incorporated in its structure. Note, however, that the latter substructures, being primarily present as pendant moieties on the macromolecule, tend to be overestimated relative to ‘core’ lignin by HSQC NMR.^53^ The HSQC data obtained are in agreement with literature.^48^

The molecular weight distribution of lignins obtained by alkaline SEC varied from 2300 Da to 3530 Da (Table 2). Independent of botanical origin, organosolv and soda lignins showed similar average *M*_w_. The molar mass of kraft lignin from softwood was the highest (3530 g mol^−1^).

The *M*_w_ values of the lignins used in this BCD study decreases in the order kraft Indulin AT > organosolv-spruce > soda-P1000 > organosolv-wheat straw > organosolv-Alcell. The molar mass distribution and dispersity are comparable to literature values.^54,55^

The syringyl (S), guaiacyl (G) and *p*-hydroxyphenyl (H) and 5-substituted phenolic end-groups, as well as aliphatic alcohols and carboxylic groups of the starting lignins were quantitatively determined by ^31^P NMR (Table 2; ESI, Fig. S12†). These results showed that lignins from non-wood, *e.g.*, straw, and from hardwood consist of all (S, G, H) phenolic units. The H-unit was found to be only in limited amounts. In contrast, the lignins derived from softwood consists of mainly G-units and some minor amount of H-units. These results therefore reflect the botanical origin of the lignins very well. Furthermore, the type of aromatic units in the starting technical lignin could be decisive to produce the type of the phenolics as is discussed hereafter in the monomer selectivity of the BCD process.

### Base catalyzed deconstruction or depolymerization (BCD) of technical lignins

**Scheme 1:**
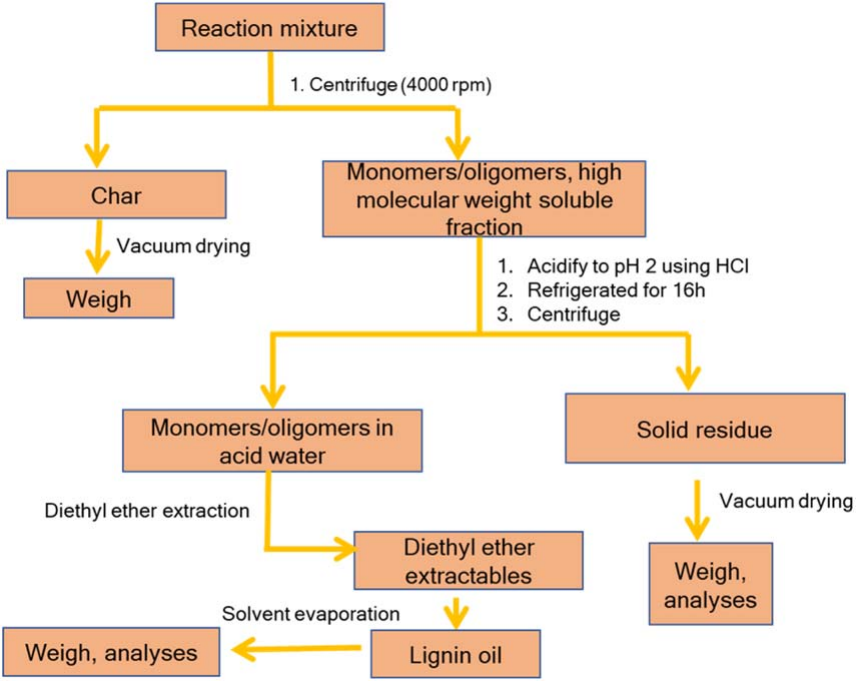
Work-up procedure after the base catalyzed depolymerization of lignin.

Considering previous reports on base catalyzed depolymerization (BCD) of lignin to obtain phenolic monomers,^6,22,23,40,42^ we decided to perform the reactions at 250 °C in a batch reactor in water using NaOH as the base catalyst under nitrogen atmosphere (40 bar). Lignin conversions and selectivity were calculated based on the definitions (eqn (1) & (2)). All BCD reactions were performed using 1.8 wt% NaOH solution in water at an initial pH of 12.7. After the reaction, lignin oil and solid lignin residue were separated by the work-up procedure depicted in Scheme 1 pH of the reaction mixture after BCD was slightly lower than 12 due to the consumption of NaOH. Before centrifugation to remove char, pH of the reaction mixture was adjusted to 12 under magnetic stirring (using 2 M NaOH) to ensure complete solubilization of residual material.

### Definitions

Lignin conversion is defined as:1
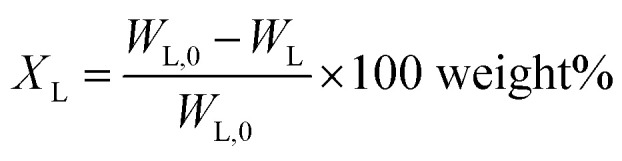
*W*_L,0_ is the initial lignin weight, *W*_L_ is the weight of the lignin residue.

Monomer yield (% w/w to lignin intake) is defined as:2
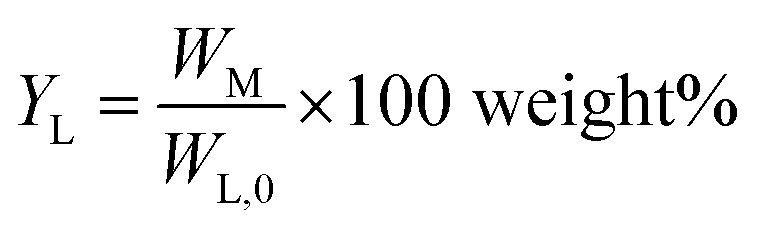
*W*_Mo_ is the total weight of monomers in the whole lignin oil.

Monomer yield (% w/w in the lignin oil) is defined as
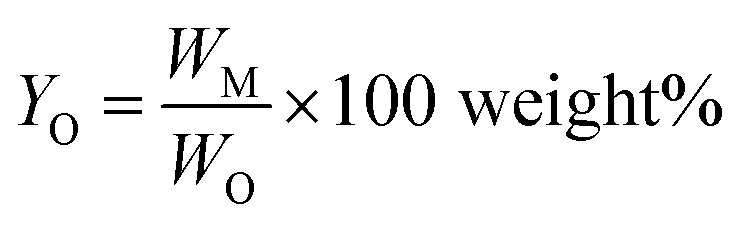
*W*_O_ is the weight of lignin oil obtained after BCD.

The lignin oils obtained were quantified for phenolic monomers using GC-FID. The results are summarized in Table 3. All BCD reactions resulted in two main product fractions, *i.e.*, a low molecular weight lignin oil and a high molecular weight base-soluble lignin residue. Char, generally accepted as polycyclic aromatic compounds obtained by the polycondensation of benzene rings^56,57^ (the base insoluble fraction), was only isolated in minor amounts (1–2% w/w). While the obtained mass balances differed for the various types of lignin tested, they are on average good to excellent for lignin depolymerization experiments and the missing part can be attributed to either gaseous products (*e.g.* CO_2_) or water-soluble components, neither of which were recovered or analyzed.

**Table 3:** Conversion and product composition of various fragments obtained by BCD^a^

Entry	Lignin	Conv. (%)	Residue (% w/w)	Char (% w/w)	Lig. oil (% w/w)	Mass balance (% w/w)	Monomer yield (Y_L_)^b^ (% w/w) to the lignin intake								Monomer yield (*Y*_O_) (% w/w) in the lignin oil^b^
							P	G	C	S	MyC	MC	EG	Total	
1	Soda-P1000	57	43	2.0	29	74	3.0	4.5	2.0	1.5	1.0	0.5	0.5	13.0	45
2	OS-Alcell	42	58	2.0	30	90	3.0	2.5	1.5	3.0	2.0	<0.5	0.5	12.5	42
3	Kraft Indulin AT	34	66	1.0	28	95	0.5	7.0	3.0	0.0	0.0	2.0	0.5	13.0	46
4	OS-spruce	30	70	1.0	20	91	1.0	6.0	2.0	0.0	0.0	1.5	0.5	11.0	55
5	OS-wheat straw	51	49	1.0	31	81	1.5	4.5	2.5	1.5	1.5	0.5	0.5	12.5	41

a Reaction conditions: lignin (1 g), 40 mL aqueous NaOH (1.8 wt%), initial pH = 12.7, *T* = 250 °C, *t* = 4 h, 900 rpm, *p*(N_2_) = 40 bar; P = phenol, G = guaiacol, C = catechol, S = syringol, MyC = methoxy-catechol, MC = methyl catechol, EG = ethyl guaiacol; OS = organosolv.

b Obtained by GC-FID.

*Note:* brisk effervescence was observed during the acidification using HCl (when the pH attained ≈4) of the reaction mixture. A plausible explanation is that CO_2_ generated during BCD reacted with NaOH to generate Na_2_CO_3_ which subsequently liberated gaseous CO_2_ during the neutralization/precipitation step with HCl.

### Conversion, oil yield, lignin residue and monomer selectivity

The data on lignin conversion, lignin oil yield and phenolic monomer composition are provided in Table 3. Lignin oil yields were approximately 30% w/w except for organosolv spruce (20% w/w). Lignin residue yields ranged from 43–70% w/w and could not be correlated to the lignin oil yields. Excellent mass balances were obtained (≥90% w/w) except for the herbaceous lignins, *i.e.* ranging from 74 to 81% w/w. All the technical lignins contained carbohydrates (Table 1), which conceivable were converted into base catalyzed water soluble products (*e.g.* lactic acid) under the prevailing reaction conditions. The low mass balance obtained with P1000 lignin is therefore attributed to the comparatively higher amounts of carbohydrates, ash and other aqueous base soluble components. Organosolv hardwood and soft wood lignin gave highest lignin residues and highest mass balances, indicating less water soluble and gaseous components compared to herbaceous lignin. Even though kraft Indulin AT was shown to contain higher abundance of HSQC detectable β-5, β-1 and β–β interunit linkages, a positive impact on BCD lignin oil and monomer yields was not observed. Most likely, the softwood/spruce lignins (composed mainly of guaiacyl units) used in this study are more resistant to BCD than a herbaceous structure suggesting large increment in C–C linkages during kraft/organosolv pulping. Hardwood derived Alcell lignin gave comparable conversion to herbaceous lignin. Given the observation that organosolv lignins prepared from hardwoods and wheat straw, similar in β-O-4 aryl ether content, yet entirely different in subunit composition, provide similar lignin oil yields indicates that BCD oil yields are primarily dictated by β-O-4 abundance.

In all cases lignin residues were the main product. Char amounts in all lignin BCD experiments at pH 12.7 were found between 1–2% w/w. This is in agreement with literature as high pH opposes the char formation with concomitant increase in lignin residue.^22,58^

Table 3 summarizes the main phenolic monomers and their yields (w/w to the lignin intake) in the lignin oil obtained by BCD at 250 °C. The phenolic monomers were firstly identified by GC-MS and then quantified by GC-FID (after silylation). The selectivity pattern of these monomers strongly depends on the difference in the population of syringyl–guaiacyl–hydroxyphenyl (SGH) structural subunits in the starting technical lignins. Guaiacol and syringol are produced as primary phenolic monomers (except in the case of softwood derived lignin) and catechol, methoxy catechol, phenol are formed *via* hydrolysis, demethylation, or demethoxylation as secondary products. Regarding soft wood lignin (Indulin AT and spruce), guaiacol was the major primary phenolic monomer and catechol was produced as the main secondary product. Spruce lignin gave lowest lignin oil yields, still total monomer yield was comparable (11% w/w to the lignin intake) to other lignins. Lowest β-O-4 linkages and relatively higher population of C–C linkages (*vide supra*, Table 2) could have decreased the production of low molecular weight oligomers in spruce.

### Characterization of lignin oils

Lignin oils were initially analyzed by conventional GC-FID followed by 2D-GC-FID measurements. The abundance of each class of aromatic compounds was determined by adding the concentration of determined compounds. The estimation of monomers by 2D-GC-FID was based on previously published reports.^20,34^ Representative 2D GC chromatograms are shown in ESI (Fig. S2–S6†). Compared to 1D-GC-FID, higher total phenolic monomer yields were observed (Table 4). The main components were phenols, methoxy phenols and dihydroxy benzenes. Cycloalkanes and other type of hydrocarbons were not detected. Some fatty acid by-products were detected (0.2–2.1%).

**Table 4:** Quantitative results from 2D-GC analysis of lignin oil from various technical lignins

Group type	Monomers (% w/w in the oil)				
	Soda-P1000	OS-Alcell	OS-wheat straw	OS-spruce	Kraft-Indulin AT
Oxygen free aromatics	0.4	0.6	0.6	0.6	0.5
Cycloalkanes	0.0	0.0	0.0	0.0	0.0
Dihydroxy benzenes	14.2	9.8	16.0	17.6	19.3
Hydrocarbons	0.0	0.0	0.0	0.0	0.0
Ketones	0.1	1.0	0.8	0.7	0.8
Methoxyphenols	20.1	22.1	21.0	30.7	28.0
Naphthalenes	0.0	0.0	0.0	0.0	0.0
Phenols	19.3	18.0	9.5	6.0	5.1
Volatile fatty acids	2.1	1.4	0.4	0.2	0.7
Total volatile fraction in oil (wt%)	54.4	52.8	48.3	55.8	54.4
Total phenolic monomers in oil (% w/w)	53.6	49.8	46.5	54.3	52.4

The total phenolic monomer contents in the lignin oil were 53.6%, 49.8%, 46.5%, 54.3% and 52.4% for soda P1000, Alcell, organosolv-wheat straw, organosolv-spruce and kraft Indulin AT respectively. Strikingly, the 2D-GC detectable monomers for the lignin oil samples are only ≈47–54 wt%, indicating the presence of non-GC-FID detectable components presumably higher molecular weight dimers/oligomers. SEC analysis of lignin oils indeed showed the presence of higher molecular weight components (*vide infra*). In order to obtain qualitative insight into these di- and oligomers, GC-MS measurements were performed after silylation. As reported previously, derivatization (silylation) of phenolic hydroxyl groups was vital to obtain the signals of higher molecular weight components.^59^ In addition to the typical phenolic monomers (phenol, methoxy phenols, catechols), higher molecular weight components (*m*/*z* = 414–570) were also observed by GC-MS in all the lignin oils from different botanical origins (ESI, Fig. S15–S17†), indicating aromatic dimeric structures.^59^ Though exact identification of these dimers was beyond the scope of the current work, the analyses did highlight both the occurrence of shared dimers amongst different lignin oils and different relative abundances of the dimers present. Silylated higher molecular weight non-volatile oligomeric structures were not detected by GC-MS.

HSQC analysis of all lignin oils clearly showed the presence of distinct aromatic signals but, typical lignin linkages were not found (ESI, Fig. S7–S11†), indicating the absence of HSQC detectable interunit lignin linkages in the released dimers/oligomers by BCD. This can be attributed to the presence of more condensed oligomeric aromatic compounds linked by non-native lignin linkages.

**Fig. 1:**
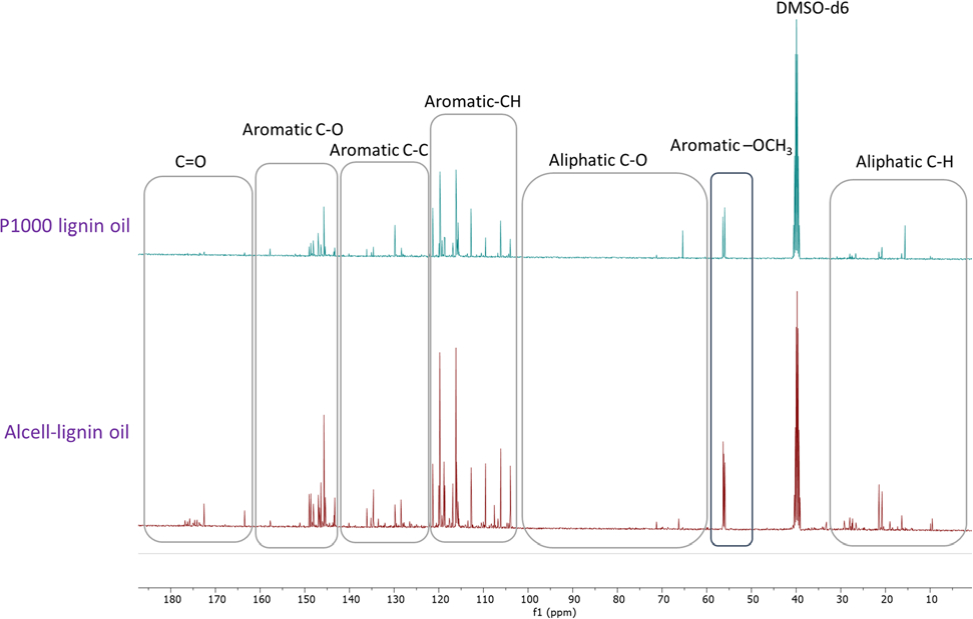
^13^C NMR spectra of soda-P1000 lignin oil and Alcell lignin oil obtained by BCD.

**Fig. 2:**
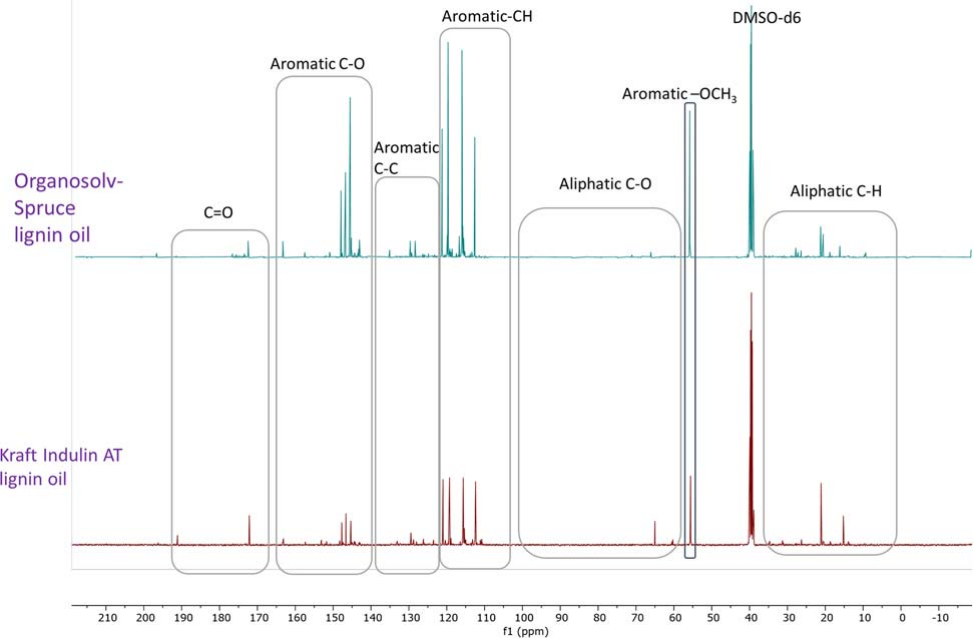
^13^C NMR spectra of organosolv softwood spruce and kraft Indulin AT lignin oil obtained by BCD.

Recently, Figueirêdo *et al.*, have shown various linkages present in pyrolytic lignin oil by 1D-^13^C NMR analysis.^60^ With reference to it, we have recorded 1D-13C NMR of all BCD lignin oils to obtain more structural insights. Lignin oils (diethyl ether soluble fraction) from all technical lignins with different botanical origins (herbaceous, hardwood and softwood lignins) were found to be rich in aromatic contents as shown in Fig. 1 and 2, as well as Fig. S14 (ESI).† The presence of aromatic –OCH_3_ groups indicates monomeric methoxy phenols and aromatic C–O indicating C–OH in phenolic units and oligomeric diaryl type components connected by C–O–C linkages (undetectable in HSQC). Irrespective of the pulping methods and botanical origin, aromatic C–C (diaryl) linkages were also observed in all lignin oils hinting towards the recombination of phenolic moieties to non-native type dimers/oligomers during BCD.

It is likely that the initial lignin breaks down during BCD releasing phenolic monomers, dimers and oligomers, and parts recombine, giving fractions of different molecular weights and solubilities.

Evidently, the recombined low molecular weight oligomeric fraction (≈50% w/w of lignin oil) is extracted to the organic solvent (diethyl ether) and accumulates in the liquid BCD oil and the higher molecular weight fraction accumulate as solid lignin residue. The tendency to recombine, however, is presumably extensively influenced by aromatic ring methoxylation. Though, a clear correlation between S/G and type of low molecular weight non-native oligomers as well as extent of lignin residue formation (the fraction where recombined higher molecular weight products likely end up), was not observed.

### Molecular weight distribution of lignin residue and lignin oil

Both lignin residues and lignin oils were analyzed by size exclusion chromatography (SEC) using 0.5 M NaOH as the eluent. All the lignin residues showed lower overall molecular weights and lower dispersity than the original lignins (Table 5). This can either suggest that the original higher molecular weight lignin was partially degraded into more uniform lower molecular weight fragments during the BCD process at 250 °C in the presence of NaOH, or that initially released monomeric or oligomeric species repolymerized. The molecular weight distribution of all the lignin oils showed a similar trend (Table 6, ESI Fig. S1†). All the lignin oil samples showed bimodal distribution of molecular weight (see ESI, Fig. S1†). The low molecular weight peak can be attributed to the phenolic monomers (phenol, methoxyphenols) and the higher molecular weight peak to oligomers (estimated degree of polymerization between 4–6) present in the lignin oil.

**Table 5:** Molecular weight distribution of lignin residue

Entry	Lignin residue	*M*_w_ (g mol^−1^)	*M*_n_ (g mol^−1^)	*Đ*
1	Soda-P1000	1830	720	2.5
2	Alcell	1960	700	2.8
3	Indulin AT	1880	810	2.3
4	Spruce	1840	680	2.7
5	Wheat straw	1850	700	2.6

**Table 6:** Molecular weight distribution of lignin oil

Entry	Lignin oil	*M*_w_ (g mol^−1^)	*M*_n_ (g mol^−1^)	*Đ*
1	Soda-P1000	790	550	1.4
		140	90	1.5
2	Alcell	870	580	1.5
		120	90	1.3
3	Indulin AT	860	530	1.6
		130	90	1.4
4	Spruce	810	470	1.7
		140	90	1.5
5	Wheat straw	840	570	1.5
		100	90	1.1

### HSQC NMR and ^31^P-NMR of lignin residues

In order to better understand the extent of depolymerization and recombination under BCD conditions, ^1^H–^13^C HSQC NMR analysis of lignin residues from all five technical lignins was performed. Despite being significantly different in initial structure, all lignin residues shared the same overall structural characteristics according to HSQC NMR analyses.

In general, the lignin residues were basically devoid of all recognizable typical lignin interunit structures (ESI, Fig. S7–S11†). Our data thus suggest that all HSQC-detectable interunit linkages, both of C–O–C as well as C–C nature, are susceptible to BCD. Interestingly, stilbene substructures might to some extent be resistant as traces remained in the lignin residues. Note, however, that in the aromatic region the signals severely overlapped and therefore assignments solely on these spectra are rather ambiguous. Despite the overlap, these analyses suggest that the aromatic subunits of the lignin residues are connected through linkages that either do not bear HSQC detectable C–H-groups or through a complex pool of low-abundance structures.

In fact, only weak signals of the typical lignin structural subunits (S, G and H units) were observed in the aromatic region of the spectra of the lignin residues, and this apparent signal suppression was only slightly improved by extensively washing the samples to remove salts likely to have accumulated during acid precipitation. Theoretically, relative to the methoxy signal, a depletion of the signals in the aromatic region (*δ*_C_ 113–102 ppm/*δ*_H_ 7.6–6.0 ppm) would point towards condensation on the G2 or S2,6 positions. Here it is important to note that neither areas were corrected for signal duplication (S units) or triplication (methoxy). Only the softwood-derived lignins showed a substantially different ratio of the aromatic and methoxy signals between the initial and lignin residues, and somewhat unexpectedly the ratio was even found to have increased (Table 7).

**Table 7:** Area quantification of aromatic to methoxy functional groups in lignin residue by HSQC NMR

Area aromatic region^a^/methoxy (%)	P1000 (soda)	Indulin AT (kraft)	Alcell (OS)	Wheat straw (OS)	Spruce (OS)
Feed lignin	37.5	34.0	46.3	41.1	39.3
Lignin residue	38.7	44.1	45.3	44.5	57.5

a *δ*_C_ 113–102 ppm/*δ*_H_ 7.6–6.0 pp.

This increase could point towards the occurrence of demethylation reactions, which can easily be conceived given the formation of monomeric catechol products, though signals corresponding to demethylated subunits could not be discerned in the spectra of the lignin residues. Indeed, ^31^P NMR showed the largest increase in phenolic OH moieties for the softwood-derived lignins, as will be elaborated below.

**Fig. 3:**
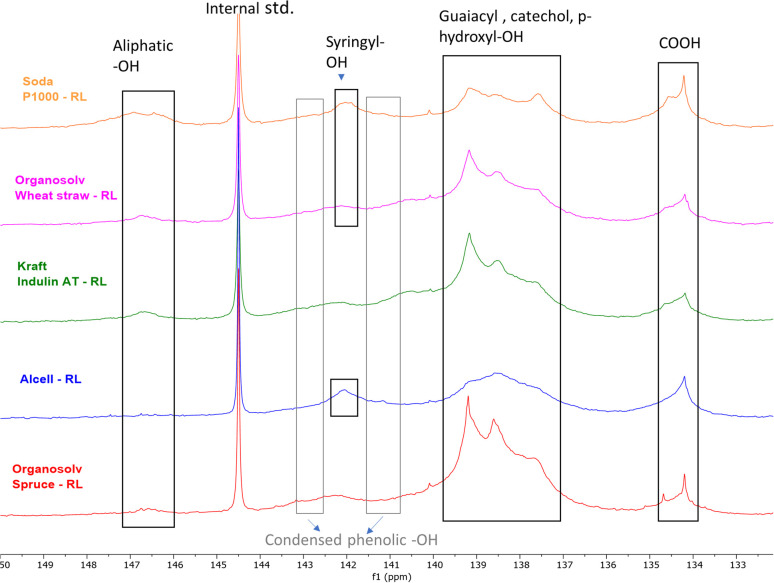
^31^P NMR (after phosphitylation) of various lignin residues (RL) obtained after base catalyzed depolymerization.

The distribution of the OH groups within the lignin residues was assessed and compared with feed lignin through ^31^P NMR (after phosphitylation) (Fig. 3 and Table 8). In general, a major decrease in aliphatic –OH groups was observed in all lignin residues pointing towards depolymerization, although this decrease may at least to some extent be attributed to the removal of carbohydrates as well. The decrease is in line with the reduction in molecular weight as observed by SEC (Table 5).

**Table 8:** ^31^P NMR data of various feed lignin and lignin residue

Lignin/lignin residue	mmol g^−1^							
	Aliphatic OH	Cond-phen OH	Syringyl OH	Guaiacyl OH	*p*-Hydroxyl OH	Guaiacyl/catechol/*p*-hydroxyl OH	COOH	Phenolic OH total (mmol g^−1^)
P1000 (soda)-feed	1.6	0.7	0.6	0.7	0.5	—	0.9	2.5
P1000 (soda)-residue	0.7	1.0	0.5	—	—	2.0	1.0	3.5
Indulin (kraft)-feed	2.1	1.3	0.0	1.6	0.2	—	0.5	3.1
Indulin (kraft)-residue	0.4	1.8	—	—	—	3.1	0.6	4.9
Alcell (organosolv)-feed	1.1	0.8	1.1	0.7	0.2	—	0.3	2.8
Alcell (organosolv)-residue	0.2	1.0	0.5	—	—	2.2	0.8	3.7
Spruce (organosolv)-feed	1.4	1.2	0	1.5	0.1	—	0.1	2.8
Spruce (organosolv)-residue	0.2	1.5	—	—	—	4.1	0.6	5.6
Wheat straw (organosolv)-feed	2.2	0.8	0.6	1.1	0.5	—	0.5	3.0
Wheat straw (organosolv)-residue	0.8	1.4	0.4	—	—	2.8	0.6	4.6

^31^P NMR of feed lignins were also measured (ESI; Fig. S12†) and different –OH functionalities were quantified.

Compared to the initial lignin samples, all lignin residues showed overlapping signals of guaiacyl (*δ* 139 ppm) and *p*-hydroxyphenyl (*δ*137.6 ppm) groups, which could indicate the presence of catechol moieties.^61^ Indeed, ^31^P NMR of a methoxy catechol standard showed that the resulting peaks coincide with those of the typical lignin subunits (ESI; Fig. S13†). Due to this overlap and broader signals, annotation is rather ambiguous. Furthermore, it should be noted that catechol moieties give double response compared to monophenols, though total aromatic hydroxyl contents are logically still truly reflected by the analysis. Furthermore, an enhancement in the condensed Ph-OH (5′ substituted) population compared to intake lignin indicates condensation of guaiacyl structural units in the lignin residue.

Organosolv-softwood spruce lignin residue showed a relatively high intensity of phenolic hydroxy signals compared to softwood kraft Indulin AT, which given its lower lignin oil yield could indicate a higher tendency of initially released methoxy groups in the guaiacyl fragments to be converted and accumulate in lignin residue as catechol moieties.

The total phenolic –OH content in herbaceous and hardwood lignin residues was also higher compared to feed lignin, again indicating aromatic polyols of higher hydroxyl number in lignin residues. ^31^P NMR data indicate that during BCD, demethylation of methoxy groups in lignin structure occurs along with lignin deconstruction. Irrespective of botanical origin, all recombined low molecular weight lignin residues showed elevated phenolic hydroxyl numbers. The combination of a reduced molecular weight and increased hydroxyl and carboxyl content likely underlies the solubility of the residual lignin at high pH.

### ^13^C and DEPT analysis of lignin residue

**Fig. 4:**
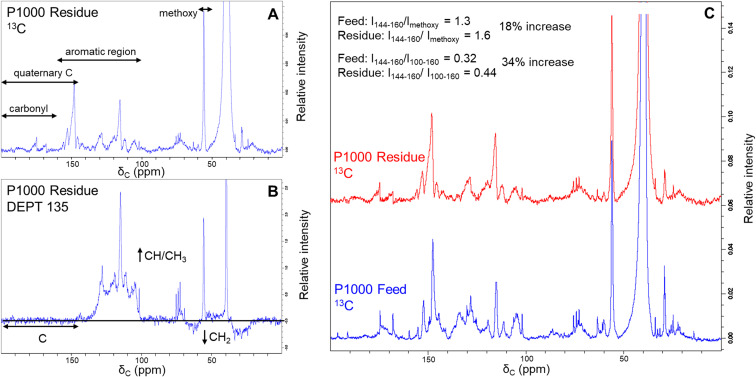
^13^C (A) and DEPT-135 (B) NMR spectra of the lignin residue obtained by BCD of soda P1000 lignin and ^13^C NMR comparison to P1000 feed lignin with semi-quantification of non-carbonyl quaternary carbons (C).

Additional evidence for structural difference between the feed lignin and residue was obtained by recording ^13^C-NMR and DEPT-135 spectra (Fig. 4). We selected soda P1000 as a typical example. DEPT 135 analysis allows distinguishing carbon multiplicity (CH/CH_3_ positive, CH_2_ negative, C silent) and thus in concert with ^13^C NMR analysis (Fig. 4A) allows for the identification of quaternary carbon groups. However, given the severe overlap of various structural motifs, the nature of these quaternary carbon atoms (*e.g.* C–C, C–O–C, and C–OH bonds) cannot be distinguished.

The DEPT135 analysis of lignin residue (Fig. 4B) clearly indicated the presence of quaternary carbon signals in the 144–200 ppm region, of which the 160–200 ppm region presumably corresponds to carbonyl functionalities. Hence, we used the signals in the non-carbonyl quaternary region (144–160 ppm) as a measure for degree of condensation of the aromatic rings in both the feed and lignin residue obtained after BCD. Relative to both the methoxy signals and aromatic regions of the ^13^C spectra, quaternary signals increased in the lignin residue compared to the feed P1000 lignin.

The intensity ratio of the quaternary signals to methoxy signals increased from 1.3 to 1.6 and that of the quaternary signals to total aromatic signals was increased from 0.32 to 0.44 in the lignin residue after BCD compared to the feed lignin. These results indicate a relative accumulation of condensed moieties in the lignin residue and thus support our previous findings that lignin residue is structurally dissimilar to feed lignin.

### Elemental analysis of lignin, lignin residue and lignin oil

C,H and O contents in the lignin, lignin residue and lignin oil were measured by elemental analysis. The results are summarized in Table S1.† It can be seen that the carbon content of the lignin residue samples slightly increased compared to the initial lignins, together with a decrease in hydrogen content, which is in line with our other observations pointing towards an overall condensation of the lignin deconstructed fragments. The oxygen content of lignin residue from all sources was lower compared to feed lignin, indicating deoxygenation (*i.e.*, demethoxylation, dehydration) to a small extent occurred in addition to depolymerization. Lignin oil compositions also showed some distinguishing characteristics. In general, all lignin oils showed lower carbon and higher oxygen contents, indicating the presence of oxygenated phenolic monomers.

### Recalcitrance of lignin residues to continued BCD

Lignin residue fractions from three main botanical origins (herbaceous, softwood, hardwood) were subjected to a second BCD reaction to validate further deconstruction to phenolic monomers. Reactions were performed using the same conditions as used for the initial BCD step. The mass balances, lignin oil yields, and phenolic monomer composition are shown in Table 9.

**Table 9:** Base catalysed depolymerization of lignin residues obtained after BCD (parantheses = data obtained from original feed lignin)^a^

Entry	Lignin residue	Conv. (%)	Residue (% w/w)	Char (% w/w)	Lig. oil (% w/w)	Mass balance (%)	Monomer yield (% w/w) to the lignin intake^b^							
							P	G	C	S	MyC	MC	EG	Total
1	Soda-P1000	28(57)	74(43)	1(2)	9(29)	84(74)	1(3)	1(4.5)	1(2)	0.3(1.5)	0.3(1)	0(0.5)	0(0.5)	3.6(13)
2	OS-hardwood Alcell	26(42)	76(58)	1(2)	8(30)	86(90)	1(3)	0.5(2.5)	0.5(1.5)	1(3)	1(2)	0(<0.5)	0(0.5)	4(12.5)
3	OS-softwood spruce	20(30)	82(70)	1(1)	6(20)	89(91)	0.3(1)	2.5(6)	1(2)	0(0)	0(0)	0.2(1.5)	0(0.5)	4(11)

a Reaction conditions: lignin residue (1 g), 40 mL aqueous NaOH (1.8 wt%), *T* = 250 °C, *t* = 4 h, 900 rpm, *p*(N_2_) = 40 bar.

b Wt% relative to lignin intake, obtained by quantitative GC-FID. P = phenol, G = guaiacol, C = catechol, S = syringol, MyC = methoxy-catechol, MC = methylcatechol, EG = ethyl guaiacol; OS = organosolv.

Irrespective of the botanical origin, all the lignin residue sample gave lower lignin oil yields and monomer selectivities compared to the initial lignins. Lignin oil yields were 9, 8 and 6% w/w for herbaceous (P1000), hardwood (Alcell) and softwood (spruce) lignin residues, respectively. Even though the characterisation of lignin residue did not show any typical lignin linkages or structural subunits, BCD of lignin residue still produces additional small amounts of phenolic monomers indicates the presence of minor “undetectable” fraction in the residual lignin amenable to cleavage. This also indicates that most of the residual lignin fraction from various technical lignin obtained by BCD are less prone to further depolymerization compared to fresh lignin under prevailing reaction conditions. Having established the abundance and recalcitrant nature of the lignin residue, future research efforts should focus on reducing its formation, thereby increasing the lignin monomer yields. However, optimizing BCD reaction conditions and reactor technologies are challenging due to the intrinsic parallel depolymerization and recondensation. Further research would be necessary to crack C–C bonds in the lignin residues. Recent promising examples of such approach involve the use of CoS_2_,^62^ Ru/NbOPO_4_ (ref. 63) and Ru/Nb_2_O_5_ (ref. 64) to cleave lignin interunit C_aromatic_–C_aliphatic_ and C_aliphatic_–C_aliphatic_ bonds.

## Conclusions

The base catalysed depolymerization (BCD) of five industrially relevant technical lignins from different botanical resources and pulping methods was systematically studied using NaOH as homogeneous catalyst in hot compressed water. Lignin oil and lignin residue fractions obtained by BCD were characterised by an extensive suite of analytical techniques. In all cases, mass balances between 74-95% w/w were obtained. The lignin oil amounts were 28–30% (w/w) for soda/organosolv herbaceous, kraft softwood, organosolv hardwood lignin. However, softwood derived spruce lignin gave only 20% (w/w) lignin oil yield, attributed to the lowest content of native interunit linkages, most amenable to cleavage. Selectivity of phenolic monomer species in the lignin oils were found to strongly relate to the subunit composition of the input lignin and hence are mainly driven by botanical origin. The main detected phenolic monomers were phenol, guaiacol, catechol, and syringol. In addition to the phenolic monomers, dimers and oligomers of varying molecular weights connected by non-native lignin linkages are also present in all the lignin oils. Independent of the lignin input, the main overall product fraction was an alkali soluble high molecular weight residue. Detailed characterisation showed that the structure of this residue is significantly different compared to the initial lignin. We established that this residue is aromatic in nature, yet more condensed, either due to a relative accumulation of initially present condensed moieties or through the formation of new bonds, and is significantly increased in total phenolic hydroxyl groups. These residual fractions exhibited a lower overall molecular weight and dispersity compared to the starting lignin. Efforts to further deconstruct the residue under BCD conditions showed that the material is significantly less susceptible to further conversion, giving low lignin oil yields with low phenolic monomer selectivities, thus confirming the more recalcitrant nature of this residue. Hence we conclude that the previously assumed unreacted lignin fraction is in fact not residual lignin, but merely a base soluble residue, as opposed to base insoluble char. Nonetheless, the increased phenolic hydroxyl groups combined with an overall decreased molecular weight and dispersity as well as higher C–C linkages, make the lignin residues interesting biopolyol and resin candidates in polymers, and the formation independent of the lignin input type guarantees widespread applicability.

## Author contributions

R. K. Pazhavelikkakath Purushothaman: conceptualization methodology, validation, formal analysis, investigation, writing original draft, visualization, writing-review & editing. G. van Erven: methodology, validation, formal analysis, investigation, writing-review & editing. D. S. van ES: writing-review & editing, supervision. L. Rohrbach: methodology, formal analysis. A. E Frissen: methodology, formal analysis. J. van Haveren: funding acquisition, Writing-review & editing. R. J. A. Gosselink: conceptualization, Writing-review & editing, supervision, funding acquisition.

## Conflicts of interest

There are no conflicts to declare.

## Supplementary Material

RA-013-D2RA06998A-s001

## References

[cit1] https://unfccc.int/process-and-meetings/the-paris-agreement/the-paris-agreement

[cit2] https://ec.europa.eu/commission/presscorner/detail/en/qanda_20_1598

[cit3] Gallezot P. (2012). Chem. Soc. Rev..

[cit4] Gallezot P. (2008). ChemSusChem.

[cit5] Alonso D. M., Bond J. Q., Dumesic J. A. (2010). Green Chem..

[cit6] Schutyser W., Renders T., Van den Bosch S., Koelewijn S. F., Beckham G. T., Sels B. F. (2018). Chem. Soc. Rev..

[cit7] Rinaldi R., Jastrzebski R., Clough M. T., Ralph J., Kennema M., Bruijnincx P. C. A., Weckhuysen B. M. (2016). Angew. Chem., Int. Ed..

[cit8] Lau M. W., Bals B. D., Chundawat S. P. S., Jin M., Gunawan C., Balan V., Jones A. D., Dale B. E. (2012). Energy Environ. Sci..

[cit9] Vanholme R., Demedts B., Morreel K., Ralph J., Boerjan W. (2010). Plant Physiol..

[cit10] Renders T., Van den Bossche G., Vangeel T., Van Aelst K., Sels B. (2019). Curr. Opin. Biotechnol..

[cit11] Haq I., Mazumder P., Kalamdhad A. S. (2020). Bioresour. Technol..

[cit12] Haile A., Gelebo G. G., Tesfaye T., Mengie W., Mebrate M. A., Abuhay A., Limeneh D. Y. (2021). Bioresour. Bioprocess..

[cit13] Li C., Zhao X., Wang A., Huber G. W., Zhang T. (2015). Chem. Rev..

[cit14] Sun Z., Fridrich B., de Santi A., Elangovan S., Barta K. (2018). Chem. Rev..

[cit15] Korányi T. I., Fridrich B., Pineda A., Barta K. (2020). Molecules.

[cit16] Brown M. E., Walker M. C., Nakashige T. G., Iavarone A. T., Chang M. C. Y. (2011). J. Am. Chem. Soc..

[cit17] Reiter J., Strittmatter H., Wiemann L. O., Schieder D., Sieber V. (2013). Green Chem..

[cit18] Chan J. C., Paice M., Zhang X. (2020). ChemCatChem.

[cit19] Olcese R. N., Lardier G., Bettahar M., Ghanbaja J., Fontana S., Carré V., Aubriet F., Petitjean D., Dufour A. (2013). ChemSusChem.

[cit20] Kloekhorst A., Heeres H. J. (2016). Catal. Sci. Technol..

[cit21] Li X., Su L., Wang Y., Yu Y., Wang C., Li X., Wang Z. (2012). Front. Environ. Sci. Eng..

[cit22] Toledano A., Serrano L., Labidi J. (2012). J. Chem. Technol. Biotechnol..

[cit23] Katahira R., Mittal A., McKinney K., Chen X., Tucker M. P., Johnson D. K., Beckham G. T. (2016). ACS Sustainable Chem. Eng..

[cit24] Valentin R. V. M. S., Thomas R., Angeliki L., Xuebing L., Lercher J. A. (2011). Chem.–Eur. J..

[cit25] Björn Rößiger R. R., Unkelbach G., Pufky-Heinrich D. (2017). Green Sustainable Chem..

[cit26] Abdelaziz O. Y., Li K., Tunå P., Hulteberg C. P. (2018). Biomass Convers. Biorefin..

[cit27] Rößiger B. , Unkelbach G. and Pufky-Heinrich D., Base-Catalyzed Depolymerization of Lignin: History, Challenges and Perspectives, in Lignin – Trends and Applications, ed. M. Poletto, IntechOpen, London, 2018, 10.5772/intechopen.72964

[cit28] Wang X., Arai M., Wu Q., Zhang C., Zhao F. (2020). Green Chem..

[cit29] Wang X., Arai M., Wu Q., Zhang C., Zhao F. (2020). Green Chem..

[cit30] Rahimi A., Ulbrich A., Coon J. J., Stahl S. S. (2014). Nature.

[cit31] Fan L., Zhang Y., Liu S., Zhou N., Chen P., Cheng Y., Addy M., Lu Q., Omar M. M., Liu Y., Wang Y., Dai L., Anderson E., Peng P., Lei H., Ruan R. (2017). Bioresour. Technol..

[cit32] Farag S., Chaouki J. (2015). Bioresour. Technol..

[cit33] Kumar C. R., Anand N., Kloekhorst A., Cannilla C., Bonura G., Frusteri F., Barta K., Heeres H. J. (2015). Green Chem..

[cit34] Kloekhorst A., Wildschut J., Heeres H. J. (2014). Catal. Sci. Technol..

[cit35] Korányi T. I., Huang X., Coumans A. E., Hensen E. J. M. (2017). ACS Sustainable Chem. Eng..

[cit36] Huang X., Atay C., Koranyi T. I., Boot M., Hensen E. J. M. (2015). ACS Catal..

[cit37] Huang X., Korányi T. I., Boot M. D., Hensen E. J. M. (2014). ChemSusChem.

[cit38] Van den Bosch S., Schutyser W., Vanholme R., Driessen T., Koelewijn S. F., Renders T., De Meester B., Huijgen W. J. J., Dehaen W., Courtin C. M., Lagrain B., Boerjan W., Sels B. F. (2015). Energy Environ. Sci..

[cit39] Erdocia X., Prado R., Corcuera M. Á., Labidi J. (2014). Biomass Bioenergy.

[cit40] Thring R. W. (1994). Biomass Bioenergy.

[cit41] Vigneault A., Johnson D. K., Chornet E. (2007). Can. J. Chem. Eng..

[cit42] Otromke M., White R. J., Sauer J. (2019). Carbon Resour. Convers..

[cit43] Rui Katahira A. M., McKinney K., Chen X., Tucker M. P., Johnson D. K., Beckham G. T. (2016). ACS Sustainable Chem. Eng..

[cit44] Erdocia X., Prado R., Corcuera M. Á., Labidi J. (2014). Biomass Bioenergy.

[cit45] Abad-Fernández N., Pérez E., Cocero M. J. (2019). Green Chem..

[cit46] Pérez E., Abad-Fernández N., Lourençon T., Balakshin M., Sixta H., Cocero M. J. (2022). Biomass Bioenergy.

[cit47] Mattsson C., Andersson S.-I., Belkheiri T., Åmand L.-E., Olausson L., Vamling L., Theliander H. (2016). Biomass Bioenergy.

[cit48] Constant S., Wienk H. L. J., Frissen A. E., Peinder P. d., Boelens R., van Es D. S., Grisel R. J. H., Weckhuysen B. M., Huijgen W. J. J., Gosselink R. J. A., Bruijnincx P. C. A. (2016). Green Chem..

[cit49] Gosselink R. J. A., Abächerli A., Semke H., Malherbe R., Käuper P., Nadif A., van Dam J. E. G. (2004). Ind. Crops Prod..

[cit50] Li H., McDonald A. G. (2014). Ind. Crops Prod..

[cit51] Lancefield C. S., Wienk H. L. J., Boelens R., Weckhuysen B. M., Bruijnincx P. C. A. (2018). Chem. Sci..

[cit52] Giummarella N., Lindén P. A., Areskogh D., Lawoko M. (2020). ACS Sustainable Chem. Eng..

[cit53] Mansfield S. D., Kim H., Lu F., Ralph J. (2012). Nat. Protoc..

[cit54] Zhu M.-Q., Wen J.-L., Su Y.-Q., Wei Q., Sun R.-C. (2015). Bioresour. Technol..

[cit55] Bauer S., Sorek H., Mitchell V. D., Ibáñez A. B., Wemmer D. E. (2012). J. Agric. Food Chem..

[cit56] Zhang C., Shao Y., Zhang L., Zhang S., Westerhof R. J. M., Liu Q., Jia P., Li Q., Wang Y., Hu X. (2020). Sci. Total Environ..

[cit57] Yang H., Dong Z., Liu B., Chen Y., Gong M., Li S., Chen H. (2021). Fuel.

[cit58] Belkheiri T., Mattsson C., Andersson S.-I., Olausson L., Åmand L.-E., Theliander H., Vamling L. (2016). Energy Fuels.

[cit59] Dao Thi H., Van Aelst K., Van den Bosch S., Katahira R., Beckham G. T., Sels B. F., Van Geem K. M. (2022). Green Chem..

[cit60] Figueirêdo M. B., Venderbosch R. H., Heeres H. J., Deuss P. J. (2020). J. Anal. Appl. Pyrolysis.

[cit61] Meng X., Crestini C., Ben H., Hao N., Pu Y., Ragauskas A. J., Argyropoulos D. S. (2019). Nat. Protoc..

[cit62] Shuai L., Sitison J., Sadula S., Ding J., Thies M. C., Saha B. (2018). ACS Catal..

[cit63] Dong L., Lin L., Han X., Si X., Liu X., Guo Y., Lu F., Rudić S., Parker S. F., Yang S., Wang Y. (2019). Chem.

[cit64] Dong L., Xia J., Guo Y., Liu X., Wang H., Wang Y. (2021). J. Catal..

