# Supplementary material for: New insights into the base catalyzed depolymerization of technical lignins: a systematic comparison
Source: RSC Adv. 2023 Feb 8;13(8):4898–909. doi: 10.1039/d2ra06998a (PMC9906982; doi:10.1039/d2ra06998a)
Supplement: RA-013-D2RA06998A-s001 [file RA-013-D2RA06998A-s001.pdf]

Electronic Supplementary Material (ESI) for RSC Advances.
This journal is © The Royal Society of Chemistry 2023

# Supporting information

## New insights into the base catalyzed depolymerization of technical lignins: A systematic comparison

Rajeesh Kumar Pazhavelikkakath Purushothaman, [a] Gijs van Erven, [a, b] Daan S. van Es, [a] Léon Rohrbach, [c] Augustinus E. Frissen, [a] Jacco van Haveren, [a] and Richard J.A. Gosselink [a]

[a.] *Wageningen Food & Biobased Research, Bornse Weilanden 9, 6708 WG, Wageningen, The Netherlands*
[b.] *Wageningen University & Research, Laboratory of Food Chemistry, Bornse Weilanden 9, 6708 WG, Wageningen, The Netherlands*
[c.] *Green Chemical Reaction Engineering, ENTEG, University of Groningen, Nijenborgh 4, 9747 AG, Groningen, the Netherlands*

### Experimental

#### Materials

Soda Protobind[TM] 1000 (mixed wheat straw/Sarkanda grass) was obtained from Green Value S.A., Switzerland. Alcell organosolv lignin (mixed hardwoods (maple, birch and poplar) was obtained from Repap Technology, Canada). Kraft Indulin AT was purchased from Ingevity, US. Spruce and wheat straw organosolv lignins were obtained by an acetone-based organosolv process developed by the Energy research Centre of the Netherlands (ECN, part of TNO)[1].

Sodium hydroxide (≥ 98%), diethyl ether (≥ 99.7%), magnesium sulfate (anhydrous, ≥ 99.5%), and hydrochloric acid (37%) were procured from Sigma-Aldrich and used as received. Bis-(trimethylsilyl) trifluoroacetamide + 1% trimethylchlorosilane reagent was purchased from Regis technologies, USA. DMF (anhydrous, 99.8%), pyridine (anhydrous, 99.8%), cyclohexanol (99%), chromium(III) acetylacetonate (99.99%), 2-chloro-4,4,5,5-tetramethyl-1,3,2- dioxaphopholane (98%), and $CDCl_3$ (99.8%D, contains 0.03% TMS) were purchased from Sigma-Aldrich.

#### Base catalyzed depolymerization (BCD) tests

Base catalyzed depolymerization of lignin was performed in a 75 mL Hastelloy C-276 batch reactor set-up (Parr Series 5000 Multiple Reactor System). The reaction was typically carried out in alkaline water at a temperature of 250°C and at 40 bar nitrogen pressure (at room temperature) for 4 h. First, the required amount of NaOH (0.4 or 1.8 w/w%) was dissolved in 40 mL deionised water. Subsequently, 1 g of lignin was added to the NaOH solution. The reactor was closed and flushed with nitrogen gas (3 times at 4 bar) and finally pressurised to 40 bar $N_2$. The reactor was heated under magnetic stirring (900 rpm). The total pressure at 250°C was 80 bars.

After the reaction, the reactor was cooled down to room temperature, depressurised, and the contents were transferred to a centrifuging tube after recording the pH. The pH was raised to 12 by the addition of 2M NaOH, if required. The char was separated by centrifugation and the alkaline soluble phase was acidified using HCl (35 %) to pH 2 to precipitate any lignin residue. The contents were stored in a refrigerator for 16 h and subsequently centrifuged to remove the lignin residue. The acidified reaction mixture was then extracted with diethyl ether (three times with three times the volume of aqueous content). The diethyl ether was dried over magnesium sulphate and subsequently evaporated using rotatory evaporator to yield the lignin oil.

Before characterization, lignin residue was washed exhaustively with deionised water to remove any residual NaCl and subsequently dried.

**Analysis**

**Chemical composition of input technical lignins**

Lignin chemical composition was determined by standard compositional analysis on the basis of previously published protocols.[2] Acid hydrolysis was performed in two stages, firstly by 72% w/w $H_2SO_4$ at 30ºC for 1 h, followed by 1M $H_2SO_4$ at 100ºC for 3h. The acid-insoluble residue was gravimetrically determined, and after ash content correction through TGA (at 900 ºC), used as measure for acid-insoluble lignin (AIL). The hydrolysate was analyzed for acid-soluble lignin (ASL) by UV-VIS spectrophotometry at 205 nm and released monosaccharides were quantified by HPAEC and expressed as anhydro monosaccharides. Ash content of the lignin was determined by TGA at 900 ºC.

**Lignin oil analysis by GC-FID and GC-MS**

The lignin oil obtained by BCD was analysed on a GC-FID (HP equipped with a flame ionization detector and a CHROMPACK capillary column of dimension 25 m x 0.32 mm x 0.30 µm). For GC-FID analysis the injector and detector temperature were set at 250 °C and 280 °C, respectively. Helium was used as the carrier gas with a flow rate of 50 mL $min^{-1}$. A split ratio of 1:25 was applied. Before analysis, the lignin oil was derivatised using a silylation procedure. Typically, 20-25 mg of lignin oil and internal standard (Bisphenol-A) were mixed thoroughly with 0.8mL of pyridine and 0.5 mL of silylating agent (Bis (trimethyl silyl) trifluoroacetamide +1% trimethyl chlorosilane, Sigma -Aldrich). This solution was kept at 70 °C for a period of 15 minutes to ensure the complete silylation and subsequently injected in the GC/GC-MS. The product identification was performed by injecting authentic standards and by GC-MS analysis on a Trace Ultra GC (Thermo Scientific) with a Restek GC column Rxi-5 ms 30 m x 0.25 mm x 0.25 µm connected to a Thermo Scientific DSQ II XL quadrupole mass selective detector (EI at 70 eV, *m/z* 35-500, 150 ms sample speed). Quantification was done based on response factors which were determined for each component by injecting known concentrations using Bisphenol-A as the internal standard.

**Lignin oil analysis by 2D-GC-FID**

GC×GC-flame ionization detection analyses were performed on all lignin oil samples with a trace GC × GC (Thermo Scientific) equipped with a cryogenic trap system and two columns: a 30 m × 0.25 mm i.d. and a 0.25 µm film of the RTX-1701 capillary column connected by using a meltfit to a 120 cm × 0.15 mm i.d. and a 0.15 µm film Rxi-5Sil MS column. To trap the sample, a dual jet modulator was applied using carbon dioxide. Helium gas at a low rate of 0.6mLmin$^{-1}$ was used as the carrier gas. Both injector and FID detector were set at 250°C.The oven temperature was 40°C for the initial 5 minutes of the analysis and then raised to 250°C at a heating rate of 3°C min.$^{-1}$ The GC pressure was set at 70kPa (at 40°C) and a modulation time of 6s was used. Before analysis, the lignin oil samples were diluted 100 times with tetrahydrofuran and 500 ppm di-*n*-butyl ether (DBE) was added as an internal standard. The quantification of lignin oil components groups was performed based on relative response factor which was previously determined by injecting known concentrations of reference components.

**Two-dimensional heteronuclear single quantum correlation NMR spectroscopy (2D-HSQC NMR)**

2D $^1H$-$^{13}C$ HSQC measurements were performed on a Bruker AVANCE III 400 MHz instrument equipped with a 5mm BBI probe with z-gradient (5 G $cm^{-1}$ $A^{-1}$). Before analysis, the sample (50 mg) was dissolved in 0.75mL of DMSO-d6 by overnight stirring. The measurements were performed using Bruker's "hsqcetgpsi2" pulse program with spectral widths of 5000 Hz (from 10 to 0 ppm) and 20843 Hz (from 165 to 0 ppm) for the $^1H$ and $^{13}C$ dimensions. The number of collected complex points was 1024

for the $^1$H-dimension with a recycle delay of 1.5 s. The number of transients was 32, and 256-time increments were always recorded in the $^{13}$C-dimension. The $^1$JCH used was 145 Hz. Processing used Gaussian apodization (GB = 0.001, LB = -0.2) in the $^1$H dimension and a squared cosine function in the $^{13}$C dimension. Prior to Fourier transformation, the data matrixes were zero-filled up to 1024 points in the $^{13}$C-dimension. The central solvent peak was used as an internal reference ($\delta_C$ 39.5 ppm; $\delta_H$ 2.49 ppm). The spectra were processed using TopSpin software. Semi-quantitative analysis of the HSQC volume integrals was performed according to Del Río et al.[3], making use of the chemical shifts reported in the literature for annotation.[4-7] (Ralph et al. 2009). $S_{2,6}$, $G_2$ and $H_{2,6}$ signals were used for S, G and H units, respectively, where S and H integrals were logically halved. $C_\alpha$-oxidized analogues were estimated in a similar manner. Tricin, *p*CA and FA were similarly estimated from their respective $T_{2',6'}$, $pCA_{2,6}$ and $FA_2$ signals, respectively. For β-*O*-4 aryl ether, β-5 phenylcoumaran, β-β resinol (divided by 2), β-1 stilbene, Z-enol ether, aryl glycerol, epiresinol and benzaldehyde substructures, their respective $C_\alpha$-$H_\alpha$ correlations were used. β-5 stilbene, dihydrocinnamyl alcohol and secoisolariciresinol substructures were semi-quantified on the basis of their $C_\beta$-$H_\beta$ correlations. $G_1$-$G_5$/$G_1$-$G_1$ and $S_1$-$G_1$/$G_5$ substructures were estimated on their $G_2$ and $S_{2,6}$ signals, with the latter being divided by 2, despite suffering from substantial signal overlap.[5, 33] Volume integration was performed at equal contour levels. Contour levels were normalized at equal size of the –$OCH_3$ integral. Amounts were calculated as a percentage of total lignin (H + G + $G_{ox}$ + S + $S_{ox}$) (per 100 aromatic rings).

**$^{31}$P-NMR analysis**

$^{31}$P-NMR measurements were performed on a Bruker AVANCE III 400 MHz instrument using a standard phosphorous pulse programme with a relaxation delay of 5 s and 256 scans. The necessary acquisition time for a good quantitative spectrum was about 25 min.

**Phosphitylation Procedure:** Stock solution of relaxation reagent (chromium (III) acetylacetonate, 3mg/mL) and internal standard (cyclohexanol, 15mg/mL) were prepared in dry pyridine. Approximately, 30 mg of dry lignin was transferred into a sample vial, dissolved in 100 µl of dry DMF and 100 µl of relaxation reagent/internal standard solution. For phosphitylation, 100 µl of phosphitylating reagent (2-chloro-4,4,5,5-tetramethyl-1,3,2-dioxaphospholane) was added to 400 µl deuterated chloroform (dry) and mixed in a HPLC vial. Then this was transferred to the lignin solution, closed and mixed and stirred for at least 10 minutes before the mixture was transferred into a 5-mm-OD NMR tube.

**$^{31}$P NMR Measurements:** $^{31}$P NMR measurements were performed on a Bruker AVANCE III 400 MHz instrument using a standard phosphorous pulse programme with a relaxation delay of 5 s and 256 scans. The necessary acquisition time for a good quantitative spectrum was about 25 min.

**Calculation:** The peak areas are calculated according to the following example (Sarkanda grass/wheat straw soda lignin, Greenvalue)

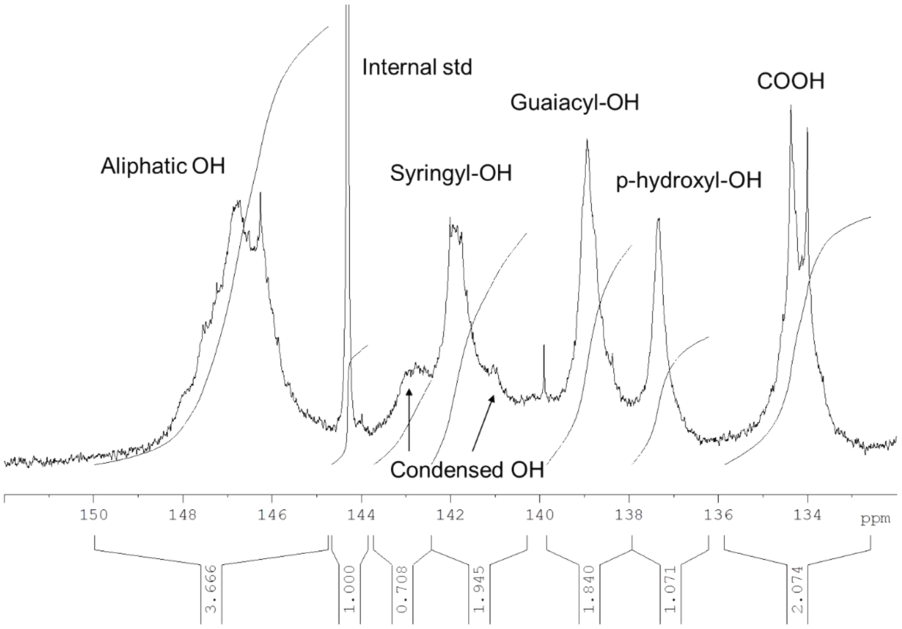


**$^{31}$P NMR integration ranges of soda-P1000 lignin after phosphitylation**

The peak attribution and area calculation is straight forward the overlapping being at a very low level. The only critical region is the one of the condensed phenolic OH that overlap with the Syringyl-OH region. The concentration of every functional group is calculated using the peak area with the IS put to equation 1

$$\text{mmol functional group/g sample} = \frac{C * A * IS}{M * L * P} \quad [1]$$

*C =* *concentration of IS solution (mg/l)*

*A =* *peak area of functional group to be calculated if area of IS =1*

*IS =* *quantity of IS added*

*M =* *molar mass of IS (g/mole)*

*L =* *dry weight of lignin (g)[corrected for moisture content]*

*P =* *purity of IS (here 0.99)*

**$^{13}$C, DEPT90 and DEPT135 NMR measurements**

$^{13}$C, DEPT90 and DEPT135-measurements were performed on a Bruker Avance III 600 MHz NMR instrument (Bruker BioSpin, Rhenstetten, Germany) equipped wit a 5 mm cryoprobe, located at MAGNEFY (MAGNEtic resonance research FacilitY, Wageningen, The Netherlands). Approximately 40 mg material was dissolved in 600 µL DMSO-d6 containing 0.01 M chromium(III) acetylacetonate ($Cr(Acac)_3$) as relaxation agent. $^{13}$C, DEPT135 ($CH/CH_3$ positive and $CH_2$ negative) and DEPT90 (CH only) NMR spectra were recorded by using the “zgig”, “deptsp135”, “deptsp90”standard Bruker pulse sequences, respectively, all by using the following parameters: spectral width -70-230 ppm, relaxation time (D1) 3.0 s, acquisition time 0.36 s, number of scans 4096, exponential apodization 20 Hz, multi-point baseline correction between 0-200 ppm applied in TopSpin 4.0.9. The central solvent peak was used as an internal reference ($\delta_C$ 39.5 ppm).

## Molar mass analyses by SEC

Alkaline SEC analyses were performed on a SEC system of Waters alliance (Waters® e2695 Separations Module) equipped with a pre-column (TOSOH Bioscience; TSK gel® $PW_{XL}$ Guard 12µm; 6.0×40mm), two serial connected columns (TOSOH Bioscience; TSK gel® $GMPW_{XL}$ Guard 13µm; 7.8×300mm) and a UV detector (Waters 2487, dual λ absorbance) operating at 280 nm. The measurements were performed at 30°C using 0.5M NaOH (Emsure® analytical reagent) as the eluent at a flow rate of 1mL/min. Poly (styrene sulphonate) sodium salts (obtained from Polymer Standard Services, GmbH) and phenol were used for the calibration. Before analyses, the samples were dissolved in 0.5 M NaOH in a 1mg/mL concentration at room temperature by gently shaking for 16h and filtered over a 0.2 um filter prior to injection.

## Elemental analysis

Elemental composition was measured with an elemental analyser (Vario Micro Cube, Elementar).

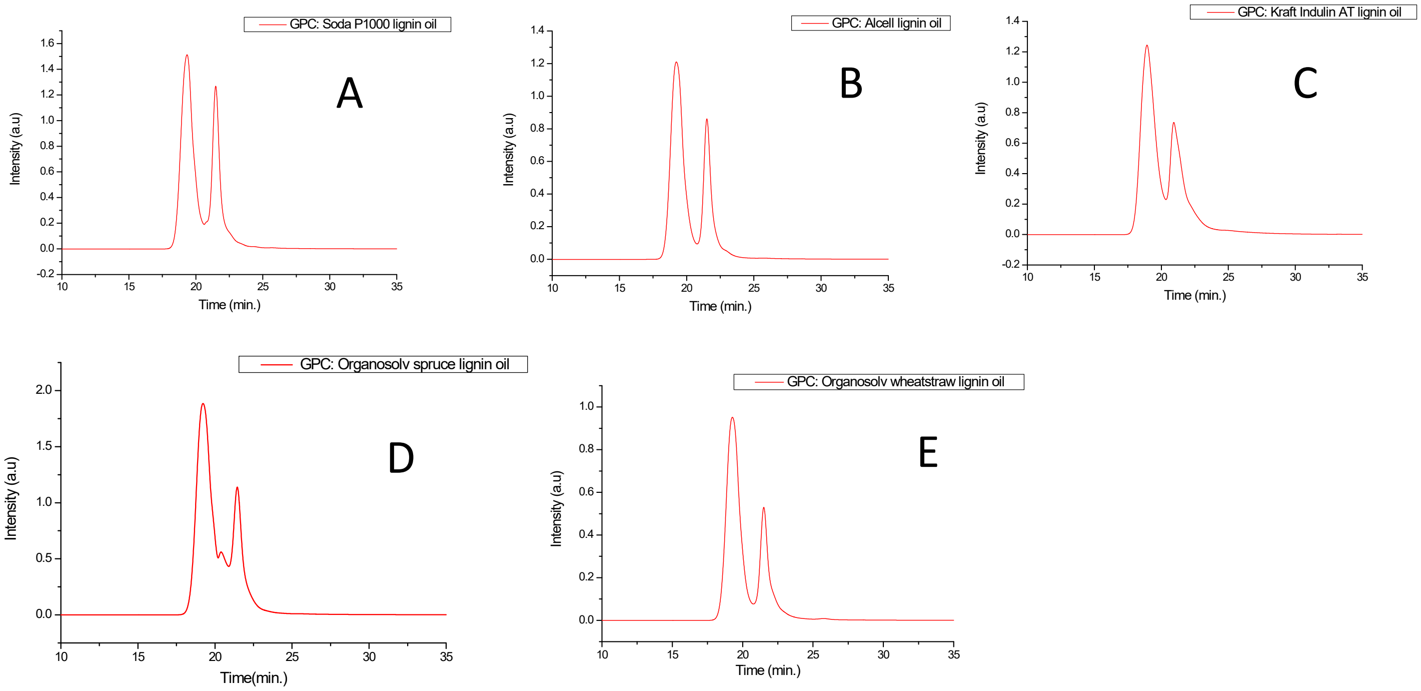


**Figure S1.** Alkaline SEC chromatogram of lignin oil obtained from (A) Soda P1000 (B) Organosolv hardwood alcell (C) Kraft Indulin AT (D) Organosolv- spruce (E) Organosolv wheatstraw

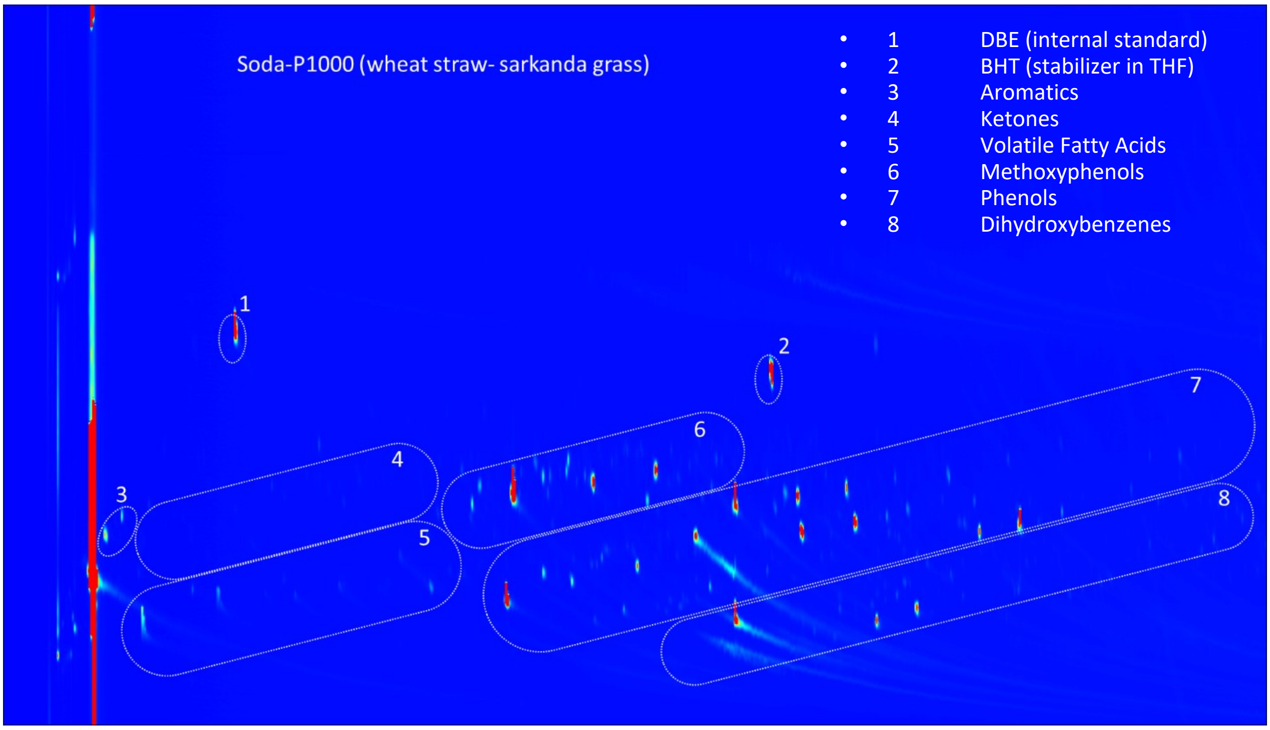


**Figure S2**. GC×GC chromatogram of the lignin oil obtained by BCD of Soda-P1000

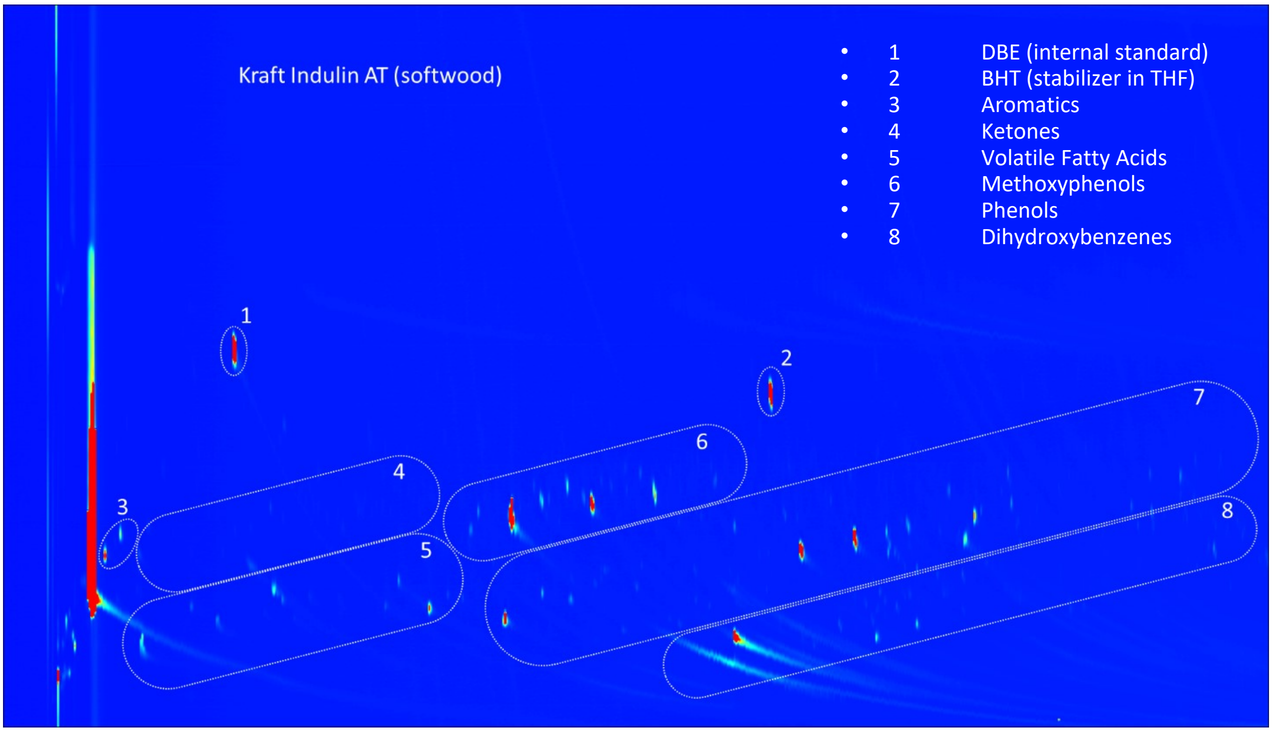


**Figure S3.** GC×GC chromatogram of the lignin oil obtained by BCD of Kraft Indulin AT

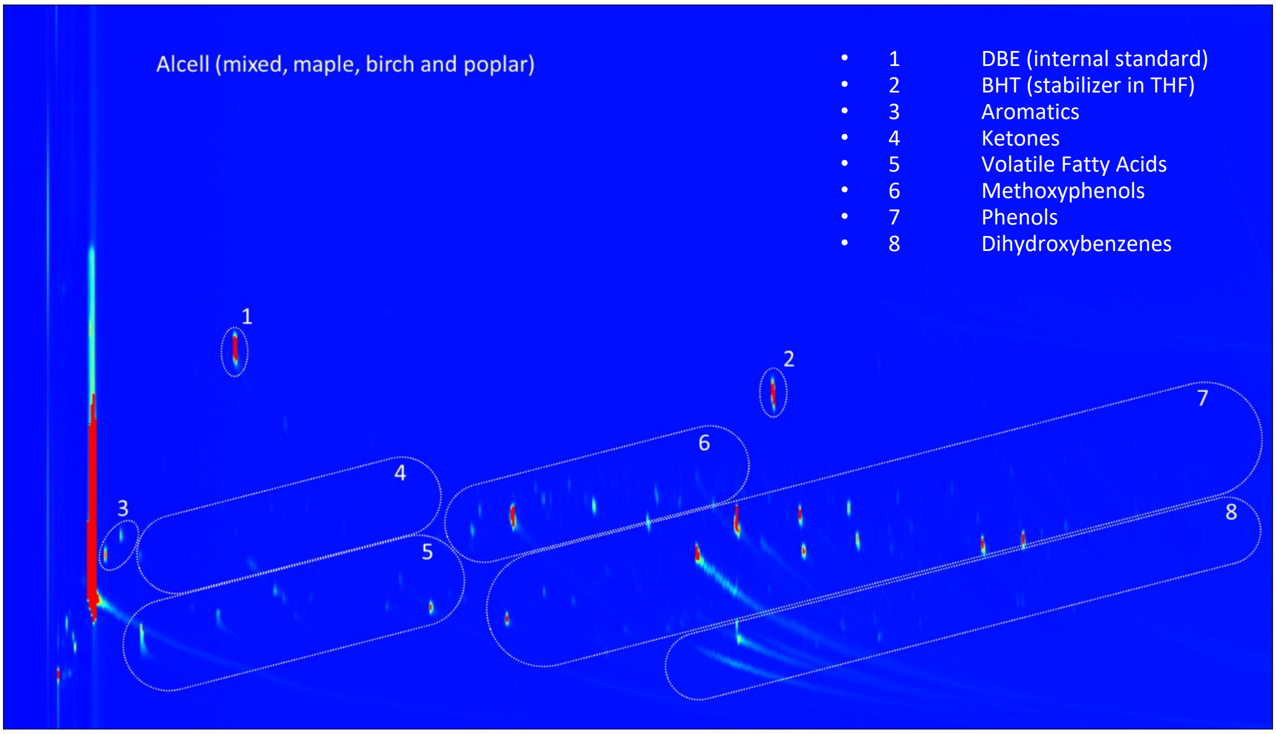


**Figure S4**. GC×GC chromatogram of the lignin oil obtained by BCD of Organosolv alcell

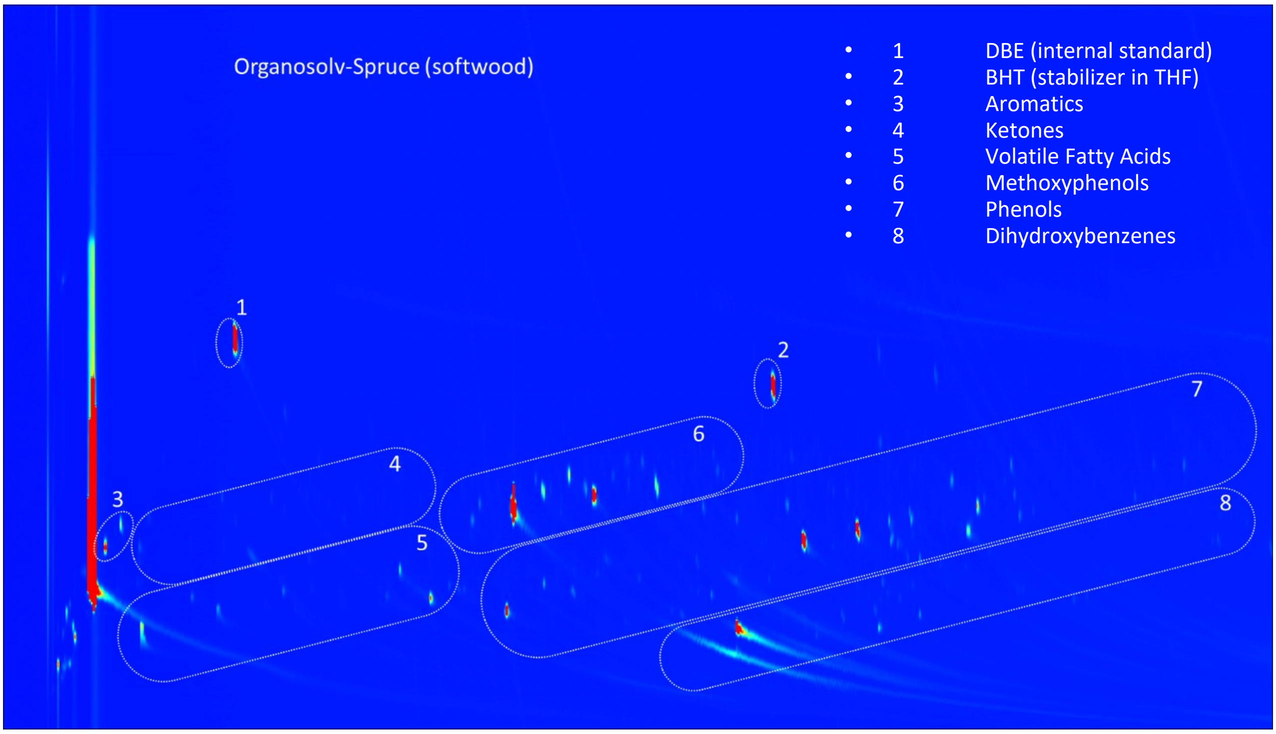


**Figure S5.** GC×GC chromatogram of the lignin oil obtained by BCD of Organosolv spruce

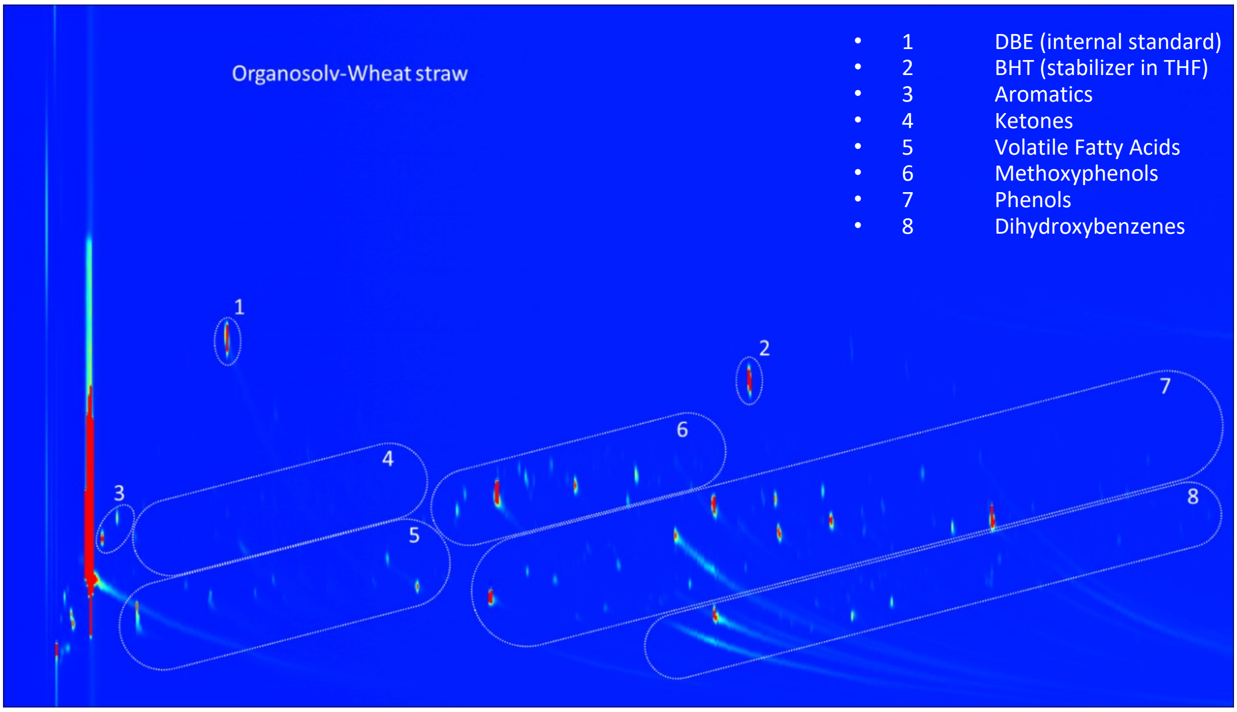


**Figure S6.** GC×GC chromatogram of the lignin oil obtained by BCD of Organosolv wheat straw

**Table S1.** Elemental (C H O) analysis of lignin, residual lignin and lignin oil

| Sample | Element (wt%) based on dry weight | | |
|---|---|---|---|
| | C | H | O |
| Soda-P1000 | 64.5 | 6.1 | 29.4 |
| Soda-P1000- lignin residue | 67.1 | 4.8 | 28.1 |
| Soda-P1000-oil | 61.8 | 6.6 | 31.2 |
| Kraft Indulin AT | 63.4 | 5.7 | 28.2 |
| Kraft Indulin AT – lignin residue | 67.7 | 5.7 | 26.5 |
| Kraft Indulin AT oil | 61.3 | 5.4 | 33.1 |
| Organosolv Alcell | 67.2 | 5.9 | 27.2 |
| Organosolv Alcell- lignin residue | 68.7 | 4.5 | 26.8 |
| Organosolv Alcell-oil | 59.3 | 5.9 | 34.5 |
| Organosolv spruce | 68.2 | 5.8 | 27.4 |
| Organosolv spruce- lignin residue | 69.8 | 4.8 | 25.4 |
| Organosolv spruce-oil | 63.7 | 6.8 | 29.5 |
| Organosolv-wheat straw | 66.3 | 6.0 | 28.2 |
| Organosolv-wheat straw- lignin residue | 67.2 | 5.0 | 27.6 |
| Organosolv-wheat straw-oil | 59.4 | 5.7 | 34.8 |

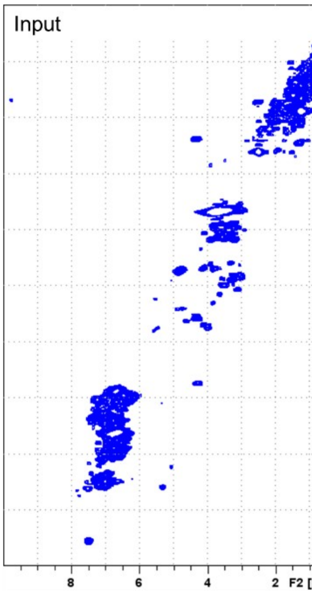


**Figure S7**. $^{1}H$-$^{13}C$ HSQC NMR

spectra of wheat straw/sarkanda grass soda P1000 lignin input, oil and residue obtained by base catalysed depolymerisation

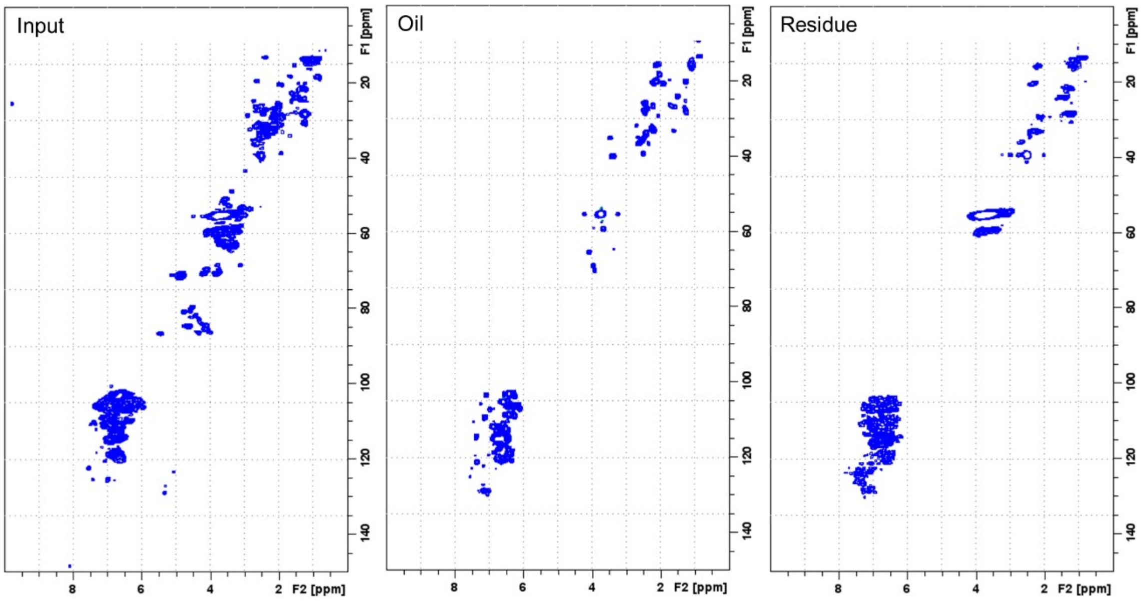


**Figure S8.**: $^{1}H$-$^{13}C$ HSQC NMR spectra of hardwood organosolv alcell lignin input, oil and residue obtained by base catalysed depolymerisation

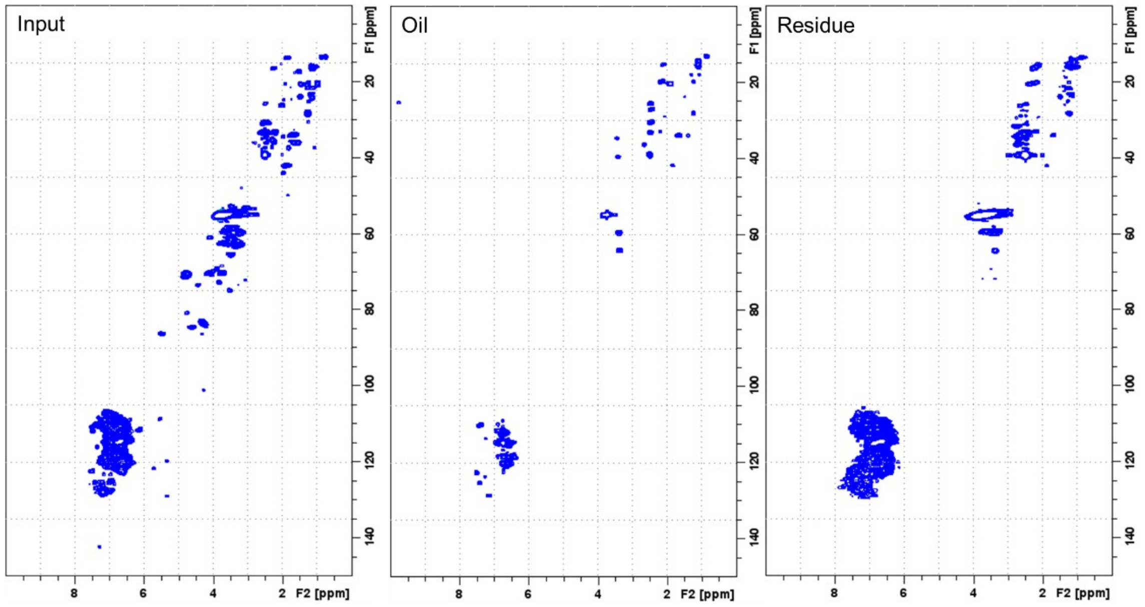


**Figure S9**. $^{1}H$-$^{13}C$ HSQC NMR spectra of softwood kraft Indulin AT lignin input, oil and residue obtained by base catalysed depolymerisation

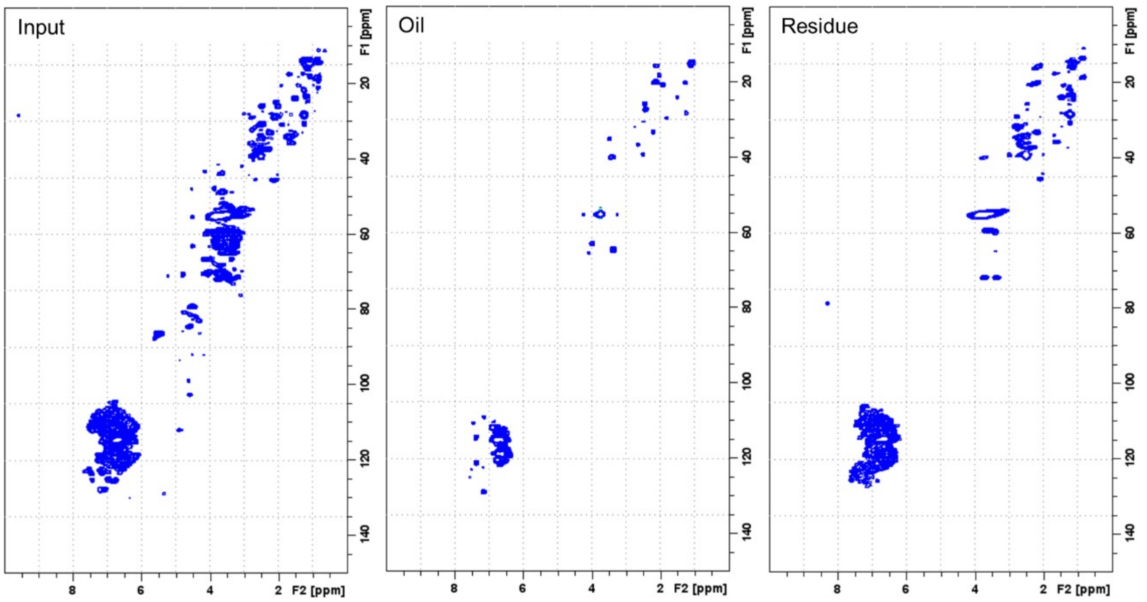


**Figure S10**. $^{1}H$-$^{13}C$ HSQC NMR spectra of organosolv softwood spruce lignin oil obtained by base catalysed depolymerisation

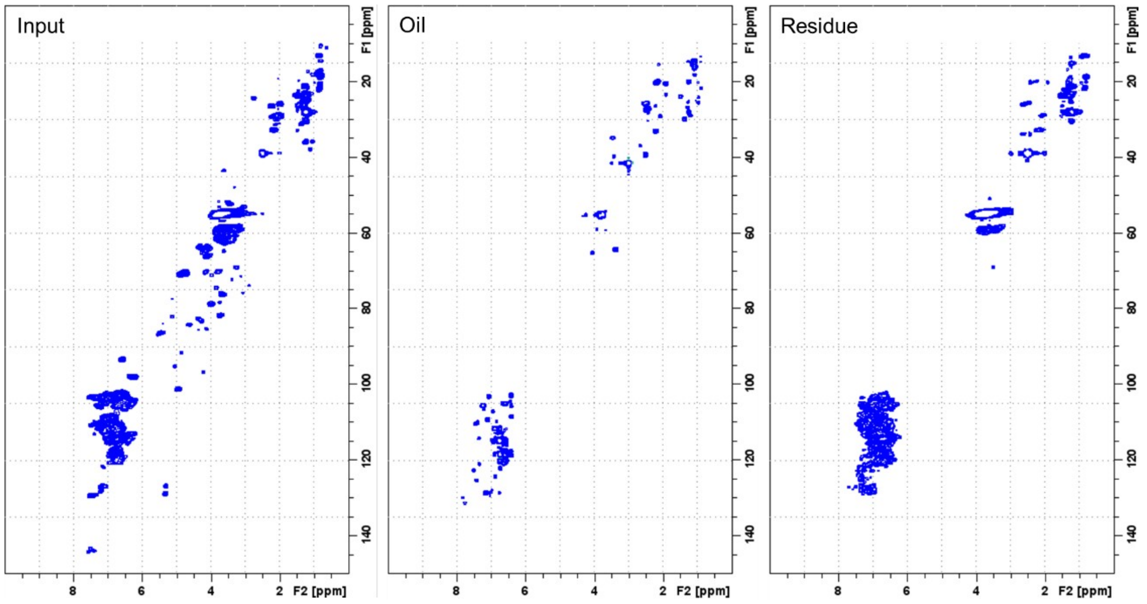


**Figure S11**. $^{1}H$-$^{13}C$ HSQC NMR spectra of organosolv wheat straw lignin oil obtained by base catalysed depolymerisation

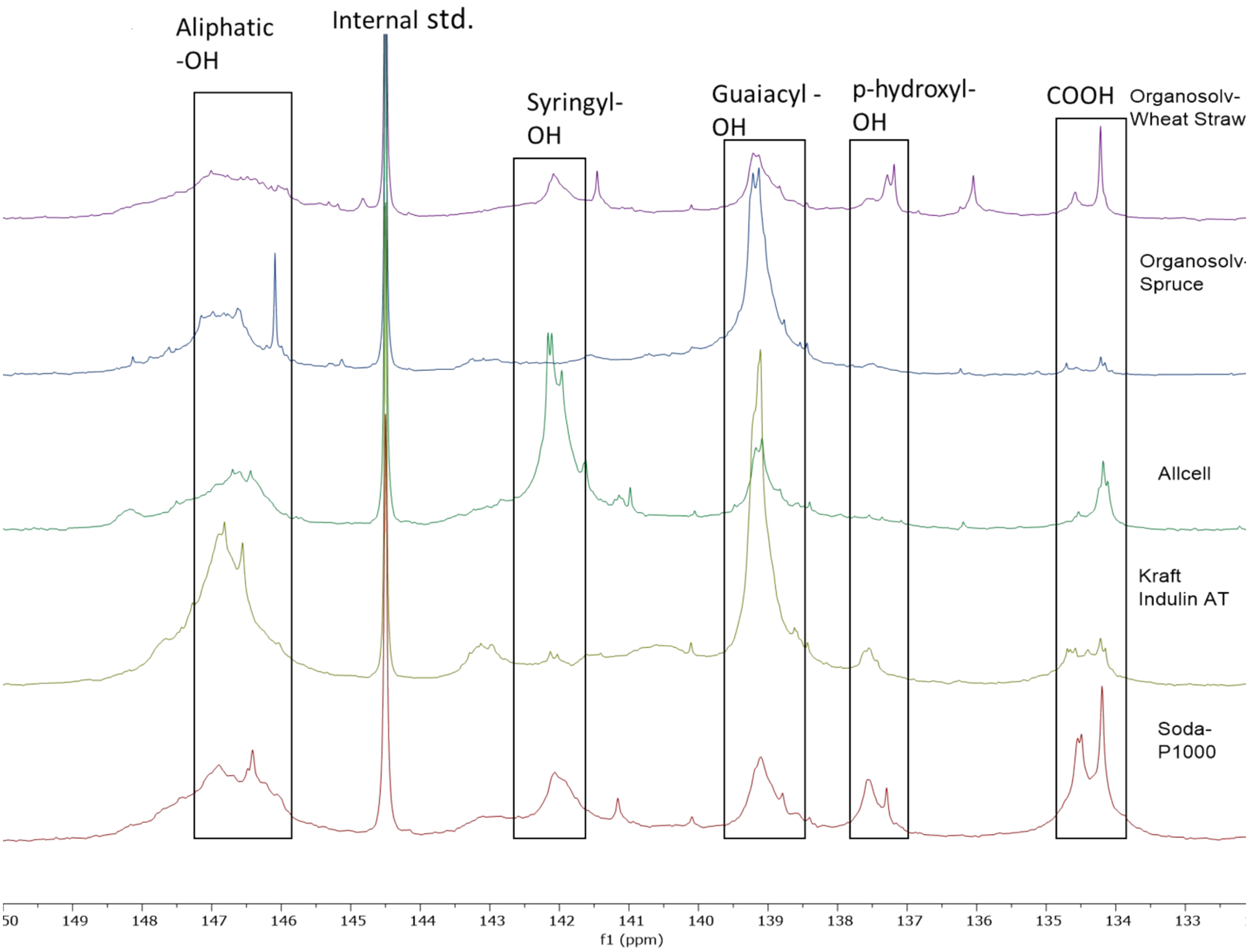


**Figure S12.** $^{31}P$ NMR (after phosphitylation) of various feed lignins

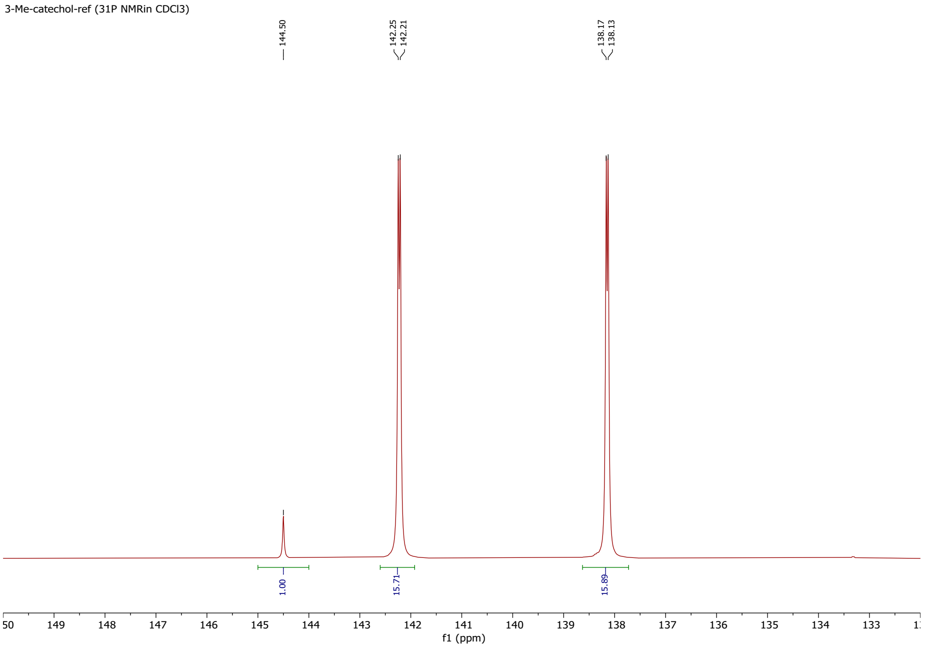


**Figure S13.** $^{31}P$NMR of reference methoxy catechol after phosphitylation

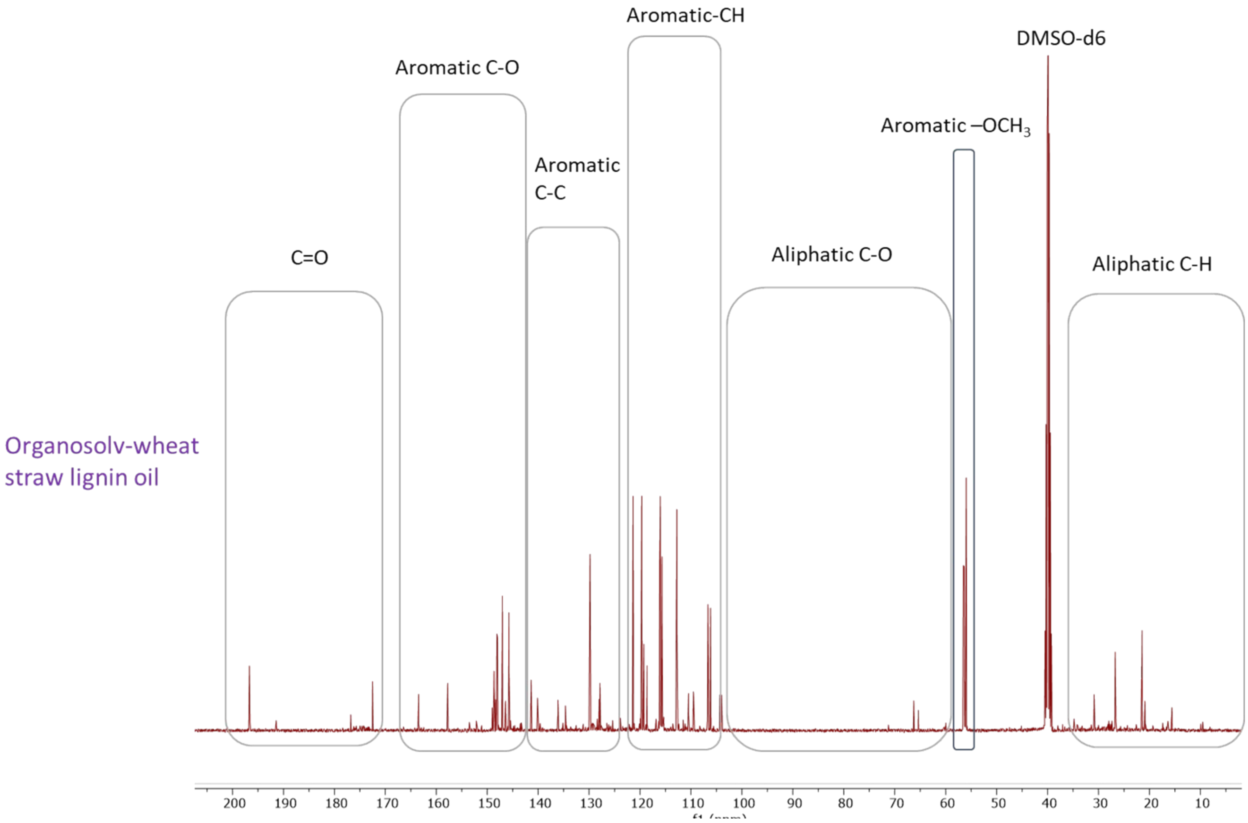


**Figure S14.** $^{13}$C NMR Organosolv wheat straw lignin oil obtained by BCD

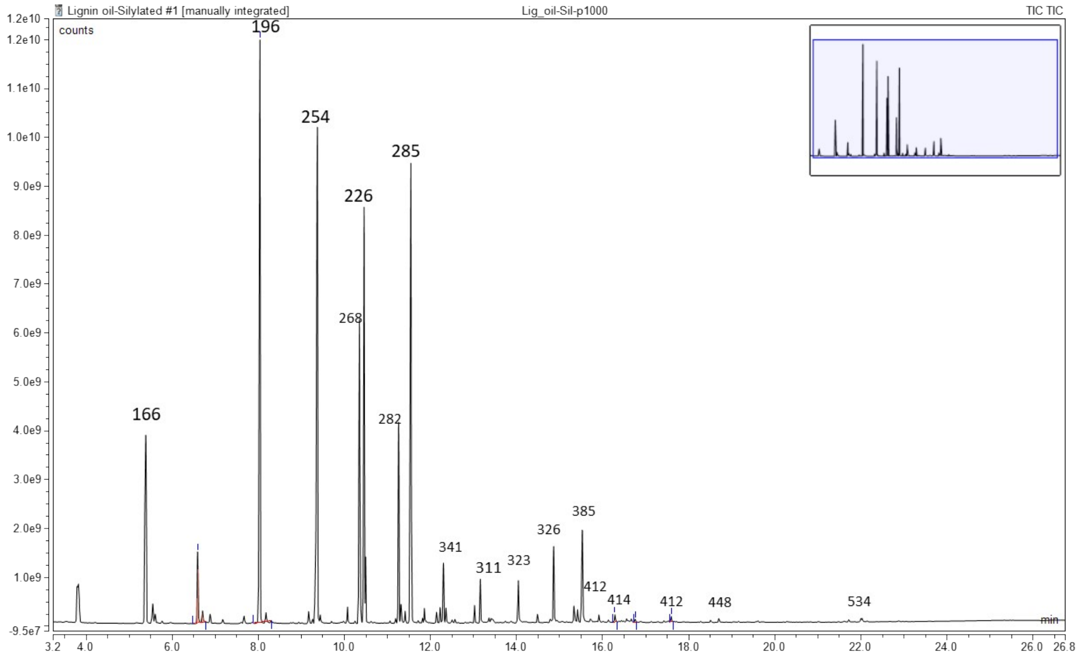


**Figure S15:** Typical GC-MS (after silylation) chromatogram of lignin oil (P1000) obtained by BCD. m/z values are provided on the top of each peak

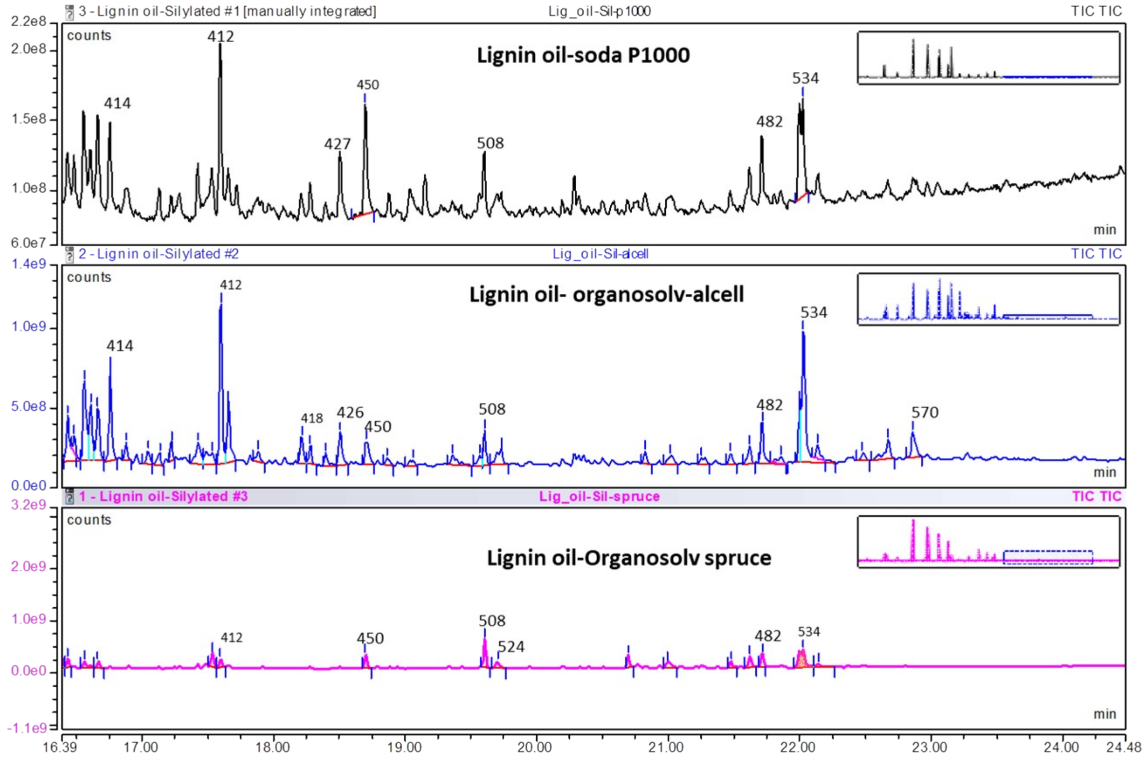


**Figure S16:** GC-MS analyses (after silylation) of lignin oil (P1000, alcell, spruce) obtained by BCD indicating higher molecular weight components and dimers ; m/z values are provided on the top of each peak

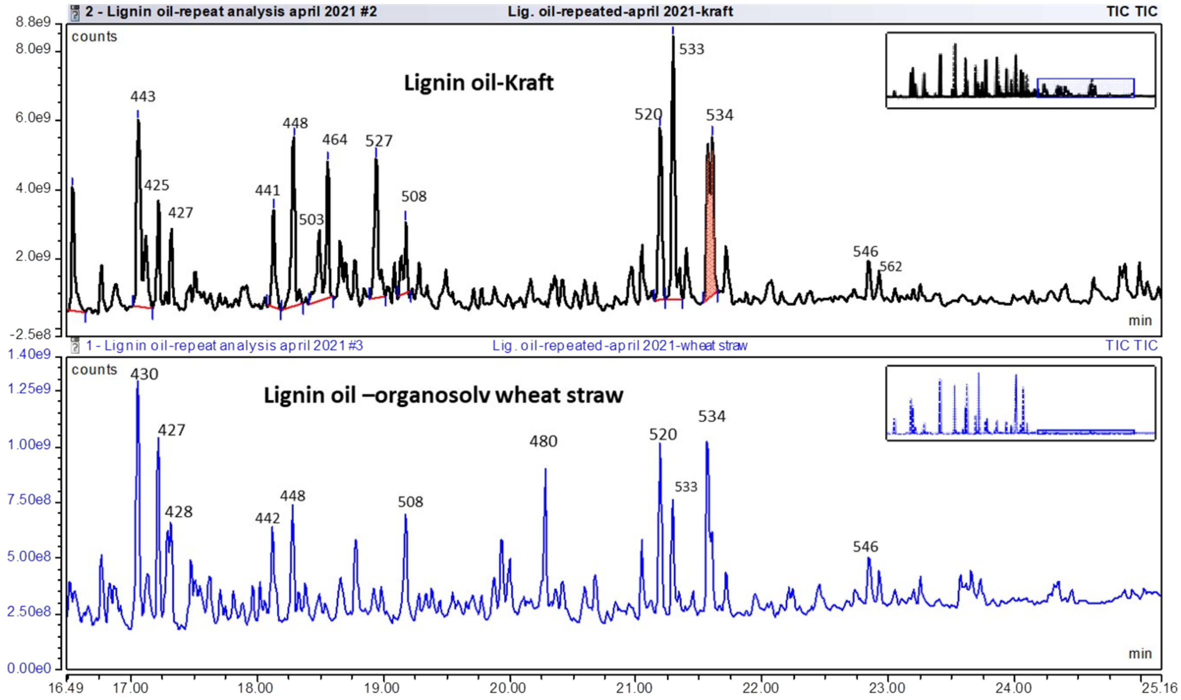


**Figure S17:** GC-MS analyses (after silylation) of lignin oil (kraft, wheat straw) obtained by BCD indicating higher molecular weight components and dimers ; m/z values are provided on the top of each peak

**References**

1. W. J. J. Huijgen, J. H. Reith and H. den Uil, *Ind. Eng. Chem. Res.*, 2010, **49**, 10132-10140.
2. S. Constant, H. L. J. Wienk, A. E. Frissen, P. d. Peinder, R. Boelens, D. S. van Es, R. J. H. Grisel, B. M. Weckhuysen, W. J. J. Huijgen, R. J. A. Gosselink and P. C. A. Bruijnincx, *Green Chem.*, 2016, 2651-2665.
3. J. C. del Río, J. Rencoret, P. Prinsen, Á. T. Martínez, J. Ralph and A. Gutiérrez, *J. Agric. Food Chem.*, 2012, **60**, 5922-5935.
4. S. A. R. Ralph, J.; Landucci, L., , *https://www.glbrc.org/databases_and_software/nmrdatabase/NMR_DataBase_2009_Complete.pdf*, 2009.
5. N. Giummarella, I. V. Pylypchuk, O. Sevastyanova and M. Lawoko, *ACS Sustain. Chem. Eng.*, 2020, **8**, 10983-10994.
6. N. Giummarella, P. A. Lindén, D. Areskogh and M. Lawoko, *ACS Sustain. Chem. & Eng.*, 2020, **8**, 1112-1120.
7. C. S. Lancefield, Hans L. J. Wienk, R. Boelens, B. M. Weckhuysen and P. C. A. Bruijnincx, *Chem. Sci.*, 2018, **9**, 6348-6360.
